# Streamlining Linear Free Energy Relationships of Proteins through Dimensionality Analysis and Linear Modeling

**DOI:** 10.1021/acs.jcim.4c01289

**Published:** 2024-12-03

**Authors:** Muhammad Irfan Khawar, Muhammad Arshad, Eric P. Achterberg, Deedar Nabi

**Affiliations:** 1 Institute of Environmental Science and Engineering (IESE), School of Civil and Environmental Engineering (SCEE), National University of Sciences and Technology (NUST), H-12, Islamabad 44000, Pakistan; 2 GEOMAR Helmholtz Centre for Ocean Research Kiel, Wischhofstr. 1-3, Kiel 24148, Germany

## Abstract

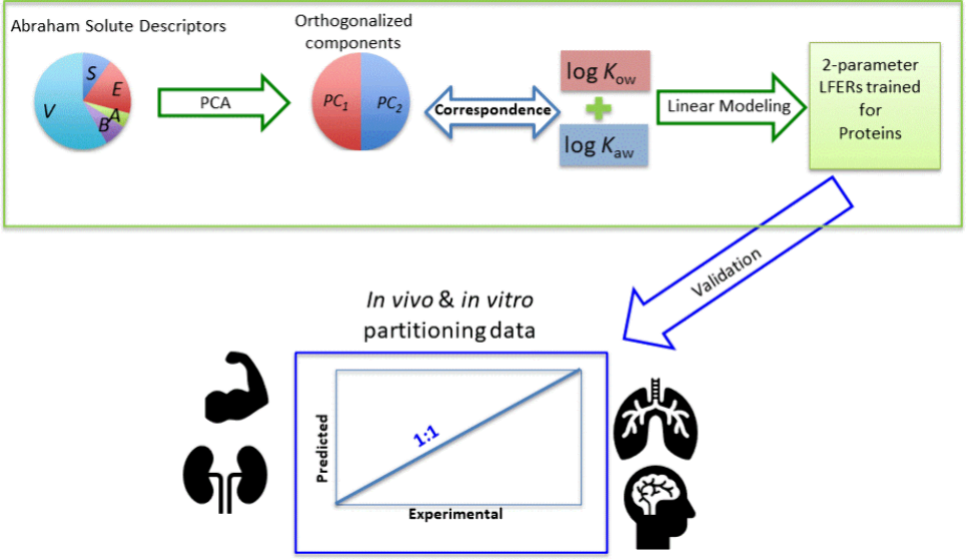

Linear free energy relationships (LFERs) are pivotal in predicting protein–water partition coefficients, with traditional one-parameter (*1p*-LFER) models often based on octanol. However, their limited scope has prompted a shift toward the more comprehensive but parameter-intensive Abraham solvation-based poly-parameter (*pp*-LFER) approach. This study introduces a two-parameter (*2p*-LFER) model, aiming to balance simplicity and predictive accuracy. We showed that the complex six-dimensional intermolecular interaction space, defined by the six Abraham solute descriptors, can be efficiently simplified into two key dimensions. These dimensions are effectively represented by the octanol–water (log *K*_ow_) and air–water (log *K*_aw_) partition coefficients. Our *2p*-LFER model, utilizing linear combinations of log *K*_ow_ and log *K*_aw_, showed promising results. It accurately predicted structural protein–water (log *K*_pw_) and bovine serum albumin–water (log *K*_BSA_) partition coefficients, with *R*^2^ values of 0.878 and 0.760 and root mean squared errors (RMSEs) of 0.334 and 0.422, respectively. Additionally, the *2p*-LFER model favorably compares with *pp*-LFER predictions for neutral per- and polyfluoroalkyl substances. In a multiphase partitioning model parametrized with *2p*-LFER-derived coefficients, we observed close alignment with experimental *in vivo* and *in vitro* distribution data for diverse mammalian tissues/organs (*n* = 137, RMSE = 0.44 log unit) and milk–water partitioning data (*n* = 108, RMSE = 0.29 log units). The performance of the *2p*-LFER is comparable to *pp*-LFER and significantly surpasses *1p*-LFER. Our findings highlight the utility of the *2p*-LFER model in estimating chemical partitioning to proteins based on hydrophobicity, volatility, and solubility, offering a viable alternative in scenarios where *pp*-LFER descriptors are unavailable.

## 1 Introduction

The partition coefficients of structural protein and albumin in water are not only crucial in pharmacokinetics^1^ but also hold significant environmental importance.^2,3^ In the field of environmental chemistry, these coefficients are essential for understanding the fate, behavior, transport, and toxicity of organic pollutants.^4^ While bioaccumulation is often considered primarily in terms of chemical accumulation in lipids, the accumulation in structural proteins, particularly for polar and hydrophilic chemicals, is also noteworthy.^2,5^ Given that structural proteins are a primary dietary source for carnivores and omnivores, the partitioning of organic chemicals into proteins may contribute to the accumulation of these chemicals through the food web. Albumin, a major component of serum proteins, has historically been used as a representative model for all protein types.^6^ However, recent studies indicate that the partitioning into albumin is significantly lower compared to structural proteins, underscoring the need to differentiate the partitioning behavior of chemicals between these two protein types.^7^ Furthermore, understanding the albumin-water partition coefficient is essential for back-calculating the freely dissolved fractions of organic chemicals in various *in vitro* cell assays,^8^ which is critical for accurately assessing chemical toxicity.

A range of techniques is used to measure the partition coefficients of structural proteins and serum albumin in aqueous environments. These include batch sorption tests,^7^ passive dosing,^7^ filtration,^9^ ultracentrifugation,^10^ and ultrafiltration.^11^ Despite these efforts, the available experimental data for these partition coefficients is restricted to just a few hundred chemicals. These experimental approaches are often labor-intensive, costly, and encounter difficulties in accurately measuring chemicals within such intricate systems. As a result, scientists frequently turn to different estimation methods for practical application of these partition coefficients.

Estimation techniques based on linear free energy relationships are commonly utilized for predicting partition coefficients. One-parameter linear free energy relationships (*1p*-LFERs), which rely on octanol–water partition coefficients,^2^ have been applied to both structural and serum proteins.^12^ However, due to octanol’s limited capacity to mimic protein properties, these methods often yield estimates within an order of magnitude for protein partition coefficients. Their accuracy diminishes particularly for chemicals with strong hydrogen bond donating characteristics, a result of octanol’s reduced sensitivity to this feature.^12^

Conversely, poly parameter linear free energy relationships (*pp*-LFERs), based on Abraham solute descriptors (ASDs), offer notably improved accuracy.^13^ These provide estimates for partition coefficients of structural^14^ and serum proteins^15^ within a two to 3-fold range. The effectiveness of *pp*-LFERs stems from their comprehensive coverage of various intermolecular interactions critical to partitioning behavior.^16^ The ASDs contain chemical information on a solute’s capacity for various types of interactions, characterized by descriptors such as *E* (polarizability/polarity), *S* (polarity), *A* (hydrogen bond donating ability), *B* (hydrogen bond accepting capacity), *V* (McGowan volume), and *L* (hexadecane-air partition coefficient, indicative of dispersion interactions).^17^ Corresponding system coefficients (*e*, *s*, *a*, *b*, *v*, and *l*) are specific to biphasic systems,^17^ such as those involving structural protein–water and serum protein–water partitioning.

The system coefficients indicate the tendencies of these phases to interact distinctively with chemicals based on the values of ASDs depicting polarizability, polarity, hydrogen bonding capabilities, molecular volume, and dispersion interactions traits of the chemicals.^17,18^ However, the broader adoption of *pp*-LFERs is currently constrained by the limited experimental database of ASDs, which encompasses fewer than 8,000 chemicals.

In our recent work, we have developed two-parameter LFERs (*2p*-LFERs), employing linear combinations of partition coefficients for octanol–water and air–water systems for various properties, including skin permeability coefficients,^19^ sensory irritation thresholds,^20^ and partition coefficients for air-blood,^21^ storage lipid–water, and phospholipid–water systems.^22^ The performance of *2p*-LFERs is on par with *pp*-LFERs and surpasses that of *1p*-LFERs. While *pp*-LFERs provide insight into partitioning behavior of compounds based on their chemical-based microscopic properties like polarizability, polarity, and hydrogen bonding,^17^ our *2p*-LFERs illuminate the partitioning behavior of chemicals in terms of macroscopic properties, such as hydrophobicity, volatility, and solubility.

The current study aims to extend the application of *2p*-LFERs beyond the aforementioned properties to encompass the partitioning properties of structural and serum proteins. Additionally, this study seeks to evaluate the efficacy of the *2p*-LFER multiphase partitioning model compared to the *pp*-LFER multiphase partitioning model in predicting *in vivo* and *in vitro* distribution ratios for various mammalian organs and tissues.

## 2 Materials and Methods

### 2.1 Data Source and Analysis

For the development and evaluation of *2p*-LFER models for structural proteins and bovine serum albumin (BSA) in water, experimental data were sourced from literature. Partition coefficients for chicken structural protein–water (log *K*_ch_, n = 46) and fish structural protein–water (log *K*_fish_, n = 45), along with bovine serum albumin-water (log *K*_BSA_, n = 83), were sourced from literature^14,15^ and detailed in Tables S1, S2, S3, and S4 of the Supporting Information (SI). Due to the absence of significant statistical differences between chicken and fish protein coefficients (Figure S1), these data were averaged to form a general structural protein–water partition coefficient (log *K*_pw_), with values ranging from 0.6 to 4.9 log units (Table S4). The values of log *K*_ow_ and log *K*_aw_ were obtained from the US EPA EPI-Suite^23^ experimental database, or, in the absence of experimental values, were estimated using *pp*-LFERs,^24^ utilizing Abraham solute descriptors from the UFZ-LSER database.^25^

### 2.2 Data Range and Diversity

In the development of robust *2p*-LFER models, priority is given not only to the data set’s size but also to its balance and representativeness. This balance is crucial for ensuring that the data set thoroughly captures a wide array of intermolecular interactions, macroscopic properties, and chemical classes. The training data sets for the *2p*-LFER models demonstrate considerable diversity in these attributes.

Specifically, the data sets for structural proteins derived from fish and chicken sources exhibit highly comparable chemical space ranges (cf. 3.1). The primary criterion for merging these data sets was to assess if the partitioning behaviors of fish and chicken proteins were sufficiently similar to combine them into a single structural protein data set, thereby expanding our *2p*-LFER model’s applicability to a broader and more diverse range of compounds. Previous research, from which these data sets were sourced, demonstrated a strong 1:1 correlation in log *K*_pw_ values, with an average absolute error of only 0.10 log units, indicating that muscle protein partitioning behavior is largely species-independent.

In addition to this literature-based justification, we performed a Bland-Altman analysis to further evaluate the compatibility of the fish and chicken data sets. This analysis (Figure S1 in the Supporting Information) showed close agreement, with only two minor outliers, reinforcing their compatibility for merging. Furthermore, as noted in Section 3.3, the regression equations for each individual data set are highly similar, supporting the representativeness of the combined data set.

By merging these data sets, we not only increased data set size but also enhanced its chemical diversity, including unique chemical classes (e.g., halogenated anilines previously absent from the fish data set). This diverse and comprehensive data set, as supported by the literature,^26^ improves model robustness and applicability across a wide range of structural proteins.

The resulting combined data set exhibits significant variability, with log *K*_pw_ values ranging from 0.6 to 4.9 log units, log *K*_ow_ values from 1.4 to 6.1 log units, and log *K*_aw_ values from −8.6 to 2.1 log units. Further analysis of the Abraham solute descriptors within the structural protein data set — specifically descriptors *E* (−0.1 to 3.63), *S* (0 to 1.98), *A* (0 to 0.69), *B* (0 to 1.28), *V* (0.79 to 1.44), and *L* (3 to 11.74) — highlights a comprehensive range of polarizability, hydrogen bonding, and dispersion forces. This suggests that the data set, as a whole, provides a full profile of molecular interaction potentials, ensuring considerable chemical diversity. The expanded data set encompasses a diverse array of chemical classes, including alkanes, haloalkanes, ethers, alcohols, ketones, substituted benzenes, phthalates, nitro compounds, and polycyclic aromatic hydrocarbons, further detailed in Table S4.

In contrast, the data set for bovine serum albumin (BSA) exhibits even greater diversity in partition coefficients. The log *K*_BSA_ values vary from 1.5 to 4.8 log units, the log *K*_ow_ values are between 1.40 and 6.8 log units, and the log *K*_aw_ values range from −10.6 to 2.2 log units (Table S6). This breadth of values for BSA covers up to 12 orders of magnitude. The set includes a spectrum of chemical classes such as alkanes, cycloalkanes, aromatic hydrocarbons, halogenated hydrocarbons, ethers, ketones, alcohols, phenols, polycyclic aromatic hydrocarbons (PAHs), and various substituted benzenes, as reported in Table S3. These classes collectively reflect the wide range of hydrophobic and hydrophilic interactions that BSA can engage in with different solutes.

### 2.3 PFAS Data and Predictive Modeling

We excluded per- and polyfluoroalkyl substances (PFAS) chemicals from the training sets but included them in a separate evaluation set to test the model’s applicability to these challenging chemicals. The partitioning data for structural proteins and BSA, available for a set of 13 ionizable PFAS,^27,28^ were employed for model evaluation (Table S7). For 47 neutral fluorotelomer compounds (Table S8), which lacked experimental log *K*_pw_ and log *K*_BSA_ values, estimates were derived using their recently published Abraham solute descriptors^29^ in corresponding *pp*-LFERs.^14,15^ For PFAS compounds, experimental values of log *K*_ow_ and log *K*_aw_ were prioritized whenever available.^30,31^ However, in cases where these values were missing, they were estimated using their ASDs^30^ in the respective *pp*-LFERs^13^ or supplemented with previously published predictions obtained through COSMO*therm*.^29^

### 2.4 Model Evaluation and Applications

Model accuracy was assessed through an indirect approach. Predicted partition coefficients for various biomolecular phases were incorporated into a multiphase equilibrium partitioning model.^12^ These contributions were normalized based on their relative abundances in a variety of mammalian organs and tissues,^12,32^ facilitating the computation of distribution ratios between plasma or blood and various organs. The generated predictions were compared against experimental *in vivo* and *in vitro* partitioning data for a set of 137 diverse compounds,^12^ spanning multiple tissues and organs across different mammalian species, including humans, rats, and rabbits (Table S9).

Furthermore, to estimate the milk-water partition coefficient, the model utilized the composition of cow milk^12^ along with the predicted coefficients for storage lipid, serum protein, and structural proteins. The validity of this approach was established by comparing the predicted milk-water partition coefficients with experimental data for 108 varied chemicals (Table S10).^12^

### 2.5 Comparative Evaluation of LFER Models

The performance of *2p*-LFERs was assessed in comparison to both *1p*-LFERs and *pp*-LFERs,^14,15^ in addition to benchmarking against experimental data. These comparisons were done not only the development of the models but also during evaluations of the models. The objective was to establish the robustness and predictive accuracy of *2p*-LFERs across varying complexities of molecular interactions.

### 2.6 Model Development and Validation

Model development and validation were carried out using R statistical environment (version 4.0.3)^33^ and XLSTAT 2020.^34^ The *2p*-LFER models were built by regressing dependent variables—log *K*_ch_, log *K*_fish_, log *K*_pw_, and log *K*_BSA_—against independent variables log *K*_ow_ and log *K*_aw_ through multiple linear regression. In selecting linear regression for our study, we closely align with the principles of LFERs,^13,35^ which elucidate a linear correlation between partition coefficients and molecular descriptors, underpinning both the predictability and theoretical precision of our approach. This methodological choice enhances the interpretability of our model, leveraging extensive data sets for log *K*_ow_ and log *K*_aw_ to forge a direct, theoretically grounded connection between chemical properties and environmental behavior. By prioritizing clear, linear relationships over the complex, nonlinear interactions typical of machine learning (ML) regressor models, our approach offers a nuanced understanding of chemical interactions, setting a distinct path that emphasizes theoretical integrity and practical applicability. Principal component analysis (PCA) quantified the necessary dimensions to encapsulate the variability present in the ASDs and assessed their relationships with log *K*_ow_ and log *K*_aw_. Pearson correlation analysis was used to investigate the interdependencies among the variables. William’s Plot, utilizing Studentized residuals and hat values, facilitated the identification of influential outliers. The robustness and predictive accuracy of the models were rigorously assessed through leave-one-out cross-validation (LOOCV), 10-fold cross-validation, and bootstrapping involving 1000 replicates (Section 1 in SI).

## 3 Results and Discussion

### 3.1 Disentangling the Complexities of *pp*-LFERs of Proteins through Dimensional Analysis

To determine whether multidimensional data sets for properties such as log *K*_ch_, log *K*_fish_, log *K*_pw_, and log *K*_BSA_, which were modeled using *pp*-LFERs based on Abraham solute descriptors (Tables S1, S2, S3, and S4), could be simplified, we conducted an analysis to assess the feasibility of representing these data sets with fewer dimensions. This approach aimed to replace the complex poly parameters with a more efficient and orthogonal set of descriptors, while maintaining accuracy. The PCA revealed that the first two dimensions accounted for significant proportions of the data set variance: 78.0% for log *K*_ch_, 78.8% for log *K*_fish_, 77.4% for log *K*_pw_, and 77.9% for log *K*_BSA_ (Figure S4 in SI), suggesting the potential effectiveness of a dimensionally reduced model. A pertinent question arising from this analysis is whether these reduced dimensions can be adequately represented by our descriptors of interest, log *K*_ow_ and log *K*_aw_.

**Figure 1:**
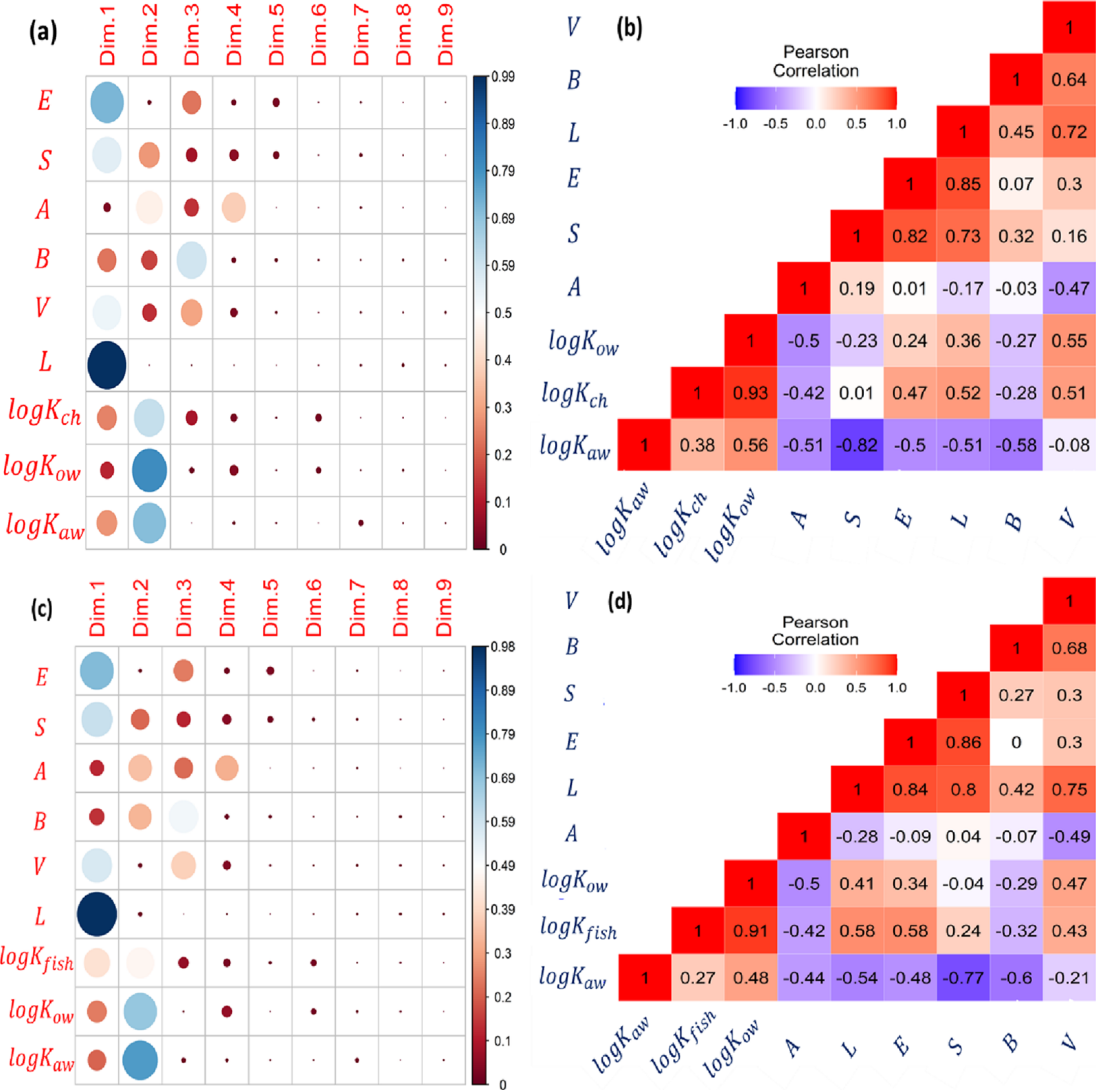
Dimensionality analyses on the calibration data sets for *2p*-LFER models of *logK*_*ch*_ and *logK*_*fish*_. The upper panels show the results obtained by (a) the Principal Component Analysis (PCA) and (*b*) Pearson Correlation Analysis performed on 46 × 9 matrix, [*logK*_*ch*_, *E*, *S*, *A*, *B*, *V*, *L, logK*_*ow*_, *logK*_*aw*_]. The lower panels show the results of (**c**) PCA and (**d**) Pearson Correlation Analysis on 45 × 9 matrix, [*logK*_*fish*_, *E*, *S*, *A*, *B*, *V*, *L*, *logK*_*ow*_*, logK*_*aw*_]. For left panels (**a**) and (**c**), the color intensity and size of the circle are proportional to the quality of presentation of a variable in each principal dimension (dim). For panels (***b***) and (**d**): each square contains value of correlation coefficient for each pair of variables. Blue and red colors show negative and positive correlations between the pairs, respectively.

To address above question, we conducted PCA on a set of variables that included the dependent variable, log *K*_pw_, along with previously employed independent variables, six ASDs, and our candidate independent variables, log *K*_ow_ and log *K*_aw_. These data sets were derived from Tables S1–S4, resulting in the creation of a 46 × 9 matrix. The analysis of the square cosine plot associated with this matrix provided key insights. It revealed that the information related to log *K*_ch_ is predominantly concentrated within the first two dimensions, with only minor contributions observed in the remaining seven dimensions (Figure 1a). In contrast, the chemical information represented by the six ASDs within this data set primarily extends to the first four dimensions, with a minor influence observed in the remaining three dimensions. This observation hints at the potential simplification of the *pp*-LFER model. Furthermore, the distribution patterns of our candidate parameters, log *K*_ow_ and log *K*_aw_, closely aligned with our property of interest, log *K*_ch_. This alignment was evident as the quality of representation of these parameters mapped well to that of log *K*_ch_. Consequently, based on this analysis, we conclude that log *K*_ow_ and log *K*_aw_ are suitable candidates for the development of a *2p*-LFER model to represent log *K*_ch_.

The validity of log *K*_ow_ and log *K*_aw_ as parameters for *2p*-LFER is further supported by the Pearson correlation analysis shown in Figure 1b. The analysis reveals a strong correlation between log *K*_ch_ and log *K*_ow_ (r = 0.93), indicating a robust linear relationship. In contrast, the correlation between log *K*_ch_ and log *K*_aw_ is moderate (r = 0.38), suggesting a weaker linear relationship. The correlation of the descriptor *E* with log *K*_ch_ is moderately positive (r = 0.47), which is noticeably higher than its correlation with log *K*_ow_ (r = 0.24). This indicates that a model based solely on log *K*_ow_ may not fully capture the polarizability spectrum of chemicals. Similarly, the variability in log *K*_ch_ attributed to the *L* parameter is not adequately described by log *K*_ow_; however, it is more closely associated with log *K*_aw_. Conversely, the McGowan volume, *V*, which is integral for representing cavity formation, does not correlate well with log *K*_aw_ in the data set, but shows a strong correlation with log *K*_ow_ (r = 0.55). The inclusion of both log *K*_ow_ and log *K*_aw_ could address these disparities in accounting for intermolecular interactions, thereby justifying the use of both parameters in the formulation of *2p*-LFER.

For the fish structural protein data set, PCA and Pearson correlation analysis on a 45 × 9 matrix, comprising log *K*_fish_, *E*, *S*, *A*, *B*, *V*, *L*, log *K*_ow_, and log *K*_aw_, revealed insights similar to those for the chicken structural protein. This similarity indicates that both protein types exhibit comparable partitioning behaviors. Consequently, when the PCA and Pearson correlation analysis was applied to a combined data set, which averaged log *K*_ch_ and log *K*_fish_ to obtain an average log *K*_pw_, resulting in a 51 × 9 matrix [log *K*_pw_, *E*, *S*, *A*, *B*, *V*, *L*, log *K*_ow_, log *K*_aw_], similar insights were observed as with the individual log *K*_ch_ and log *K*_fish_ matrices. This observation justifies the merger of the two data sets in the analysis.

In the study of bovine serum albumin protein, an 83 × 9 matrix encompassing variables such as log *K*_BSA_, *E*, *S*, *A*, *B*, *V*, *L*, log *K*_ow_, and log *K*_aw_ was subjected to PCA and Pearson Correlation Analysis. This analysis shed light on significant patterns. Predominantly, log *K*_BSA_ was found to be represented within the first two dimensions, showing a notable alignment with the distributions of log *K*_ow_ and log *K*_aw_. The correlation of log *K*_BSA_ with log *K*_ow_ was relatively strong (r = 0.87), while its correlation with log *K*_aw_ was much weaker (r = 0.06), differing from the patterns observed in the structural protein data. Notably, the *S* descriptor demonstrated a more significant correlation with log *K*_BSA_ (r = −0.22) than with log *K*_ow_ (r = −0.01), indicating that log *K*_ow_ alone may not suffice to represent the full spectrum of chemical polarity. Furthermore, variations in log *K*_BSA_ related to *E*, *L*, and *B* were better correlated with log *K*_aw_ than with log *K*_ow_. Therefore, incorporating both log *K*_ow_ and log *K*_aw_ into the model appears justified, as it could rectify these discrepancies, affirming their utility in the formulation of a *2p*-LFER.

### 3.2 *2p*-LFER Models

In this section, we describe the results of 2p-LFER models, which were obtained with the input of logK_ow_ and logK_aw_ for the estimation of structural protein–water and albumin-water partition coefficients for neutral organic chemicals.

### 3.3 Structural Protein–Water

The effectiveness of *2p*-LFER models is well demonstrated through the analysis of data sets pertaining to chicken structural protein, fish structural protein, and combined structural protein. These models, employing a linear combination of log *K*_ow_ and log *K*_aw_, provide an understanding of partitioning behavior across various structural protein types. The parameters, log *K*_ow_, indicative of hydrophobicity, and log *K*_aw_, a ratio reflecting volatility to solubility, are pivotal in elucidating the partitioning dynamics.

For chicken structural protein, the *R*^2^ value is 0.882, and the Adj.*R*^2^ is 0.877, indicating a robust linear relationship between the combined effects of log *K*_ow_ and log *K*_aw_ and the partitioning behavior. This trend persists in the fish structural protein and combined structural protein data sets, with *R*^2^ values of 0.870 and 0.878, and Adj.*R*^2^ values of 0.864 and 0.873, respectively. The consistently similar *R*^2^ and Adj.*R*^2^ values across all data sets signify the adeptness of the models in capturing the underlying linear relationships, demonstrating the robustness of the *2p*-LFER approach.

The exclusion of log *K*_ow_ from LFER resulted in a substantial decrease in explained variance, with an *R*^2^ of 0.119, underscoring the variable’*s* significant predictive contribution. Conversely, omitting log *K*_aw_ from LFER yielded an *R*^2^ of 0.844, indicating that log *K*_ow_ alone maintains considerable predictive power. These findings underline the importance of log *K*_ow_ as a key factor in determining log *K*_pw_. However, the dimensionality analysis discussed in the previous section highlights the relevance of log *K*_aw_ for certain chemicals, particularly those with specific polar intermolecular interactions. Therefore, to achieve optimal predictive accuracy, it is essential to include both log *K*_ow_ and log *K*_aw_ in the model.

The RMSE values — 0.323 for chicken structural protein, 0.353 for fish structural protein, and 0.334 for combined structural — underscore the accuracy of models. These low RMSE values indicate that the *2p*-LFER models yield predictions closely aligned with observed data, signifying minimal prediction errors and reinforcing the practical applicability of models.

Moreover, the standard errors (SE) associated with the fitting coefficients for log *K*_ow_ and log *K*_aw_ in each data set provide insights into the precision of the models. The relatively low SE values for these coefficients in all data sets emphasize the accuracy of the estimates, affirming their statistical significance. This accuracy in coefficient estimation enhances the credibility of the *2p*-LFER models, underscoring their effectiveness in capturing the combined influence of hydrophobicity and the balance between volatility and solubility in protein–water partitioning.

In summary, the statistical parameters — *R*^2^, Adj.*R*^2^, RMSE, and SE — across the data sets solidify the capabilities of the *2p*-LFER models in linear fitting, prediction accuracy, and the statistical significance of the coefficients. These models emerge as robust tools for understanding and predicting partitioning behavior in various structural proteins, highlighting the power and versatility of the *2p*-LFER approach in protein–water interaction studies.

The efficacy of model was further evaluated through cross-validation techniques. *k*-fold (5 folds) and repeated *k*-fold (5 folds, 10 repeats) cross-validation yielded mean scores of 0.838 and 0.811, respectively, which corroborates the robustness and predictive capability of the model. Bootstrap validation, with a high mean score of 0.876 and a low standard deviation of 0.043, reinforces the stability of model across various resampled subsets of data.

The hold-out method, utilizing a 20:80 test-train split (Tables S11 and S12), produced a similar model with an eq 1:
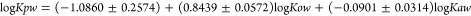
1

This model demonstrated a strong predictive performance on the test data, with an *R*^2^ value of 0.884 and an RMSE of 0.276, indicating a slightly better fit than the model derived from the full data set.

These collective findings from the main model and various validation techniques indicate a high level of model reliability and generalizability. The consistent *R*^2^ values across the main and hold-out models suggest that a significant proportion of the variance in log *K*_pw_ is systematically captured by the predictors in different subsets of data. The RMSE values further support the model’s precision in predicting new data. The consistency in the performance metrics across the full data set and the validated models underscores the model’s potential applicability in practical scenarios, marking it as a valuable tool for biochemical and environmental partitioning studies.

The comparative analysis of log *K*_ow_ and log *K*_aw_ in Table 1 illuminates their distinct roles in influencing log *K*_ch_, log *K*_fish_, and log *K*_pw_. The predominance of log *K*_ow_ reflects its substantial impact on hydrophobic interactions in protein–water partitioning, whereas log *K*_aw_, though significant, exhibits a relatively lesser influence, capturing the interplay between volatility and solubility in the partitioning process.

**Table 1:** Fitting Coefficients and Regression Statistics of *2p*-LFER Model Equation^a^ for Protein and Lipid Phases

phase	λ_1_ (±SE)^b^	λ_2_ (±SE)	λ_3_ (±SE)	R^2^	**Adj. R**^**2**^	F-statistics	RMSE^c^	source
chicken structural protein	0.813 (±0.049)	–0.077 (±0.024)	–0.874 (±0.223)	0.882	0.877	161.7	0.323	current Study
fish structural protein	0.886 (±0.055)	–0.097 (±0.027)	–1.243 (±0.246)	0.870	0.864	140.8	0.353	current Study
combined structural proteins	0.851 (±0.049)	–0.092 (±0.025)	–1.080 (±0.220)	0.878	0.873	173.3	0.334	current Study
bovine albumin serum protein	0.788 (±0.046)	–0.053 (±0.018)	0.000^d^	0.760	0.759	130.5	0.422	current Study
phospholipid	1.070 (±0.021)	– 0.056 (±0.013)	–0.247 (±0.095)	0.953	0.952	1293	0.414	Khawar et al.^22^
storage lipid	1.102 (±0.016)	0.069 (±0.01)	–0.236 (±0.043)	0.971	0.970	5046	0.375	Khawar et al.^22^

a Mathematical form of *2p*-LFER equation: log*K*_*phase*–*water*_ = λ_1_*K*_*ow*_ + λ_2_*K*_*aw*_ + λ_3_.

b The standard error (SE) represents a 95% confidence interval for the fitted values, estimated through 1000 synthetic resamples using the bootstrap method.

c RMSE: Root Mean Squared Error.

d A value of 0.000 signifies that the fitted coefficient was statistically equivalent to zero. Consequently, the related parameter was excluded and the regression analysis was repeated.

The similarity in the fitting coefficients and regression statistics of the *2p*-LFER model equations across the chicken, fish, and combined data sets (Table 1) suggests a remarkable consistency in the partitioning behavior of these structural proteins. This consistency justifies integrating their data sets to develop a more generalized understanding of structural protein–water partitioning. Such an integrated approach streamlines predictive modeling for various structural types, enhancing the practical utility of these findings in the field.

The comparison of partitioning behaviors among structural proteins, storage lipids, and phospholipids in terms of hydrophobicity and a proxy parameter (log *K*_aw_) for volatility and solubility of chemicals is intriguing. This analysis can be conducted by examining the fitting coefficients of *2p*-LFERs for structural proteins and comparing them with those from previously established *2p*-LFERs for storage lipids and phospholipids.^22^ The *2p*-LFER equations for these three phases reveal that the octanol–water partition coefficient (log *K*_ow_) positively influences the partitioning behavior of chemicals across all three phases, including storage lipids, phospholipids, and proteins. However, the storage lipids and phospholipids exhibit approximately twice the hydrophobic interaction compared to protein.

The role of the log *K*_aw_ varies among the three phases. For storage lipids, an increase in log *K*_aw_ correlates with greater partitioning into the lipid phase. In contrast, for phospholipids and proteins, a higher log *K*_aw_ is associated with reduced partitioning into these phases. Furthermore, the influence of log *K*_aw_ is marginally more pronounced in proteins than in storage lipids and phospholipids, as indicated by the relative magnitudes of the fitting coefficients for log *K*_aw_.

**Figure 2:**
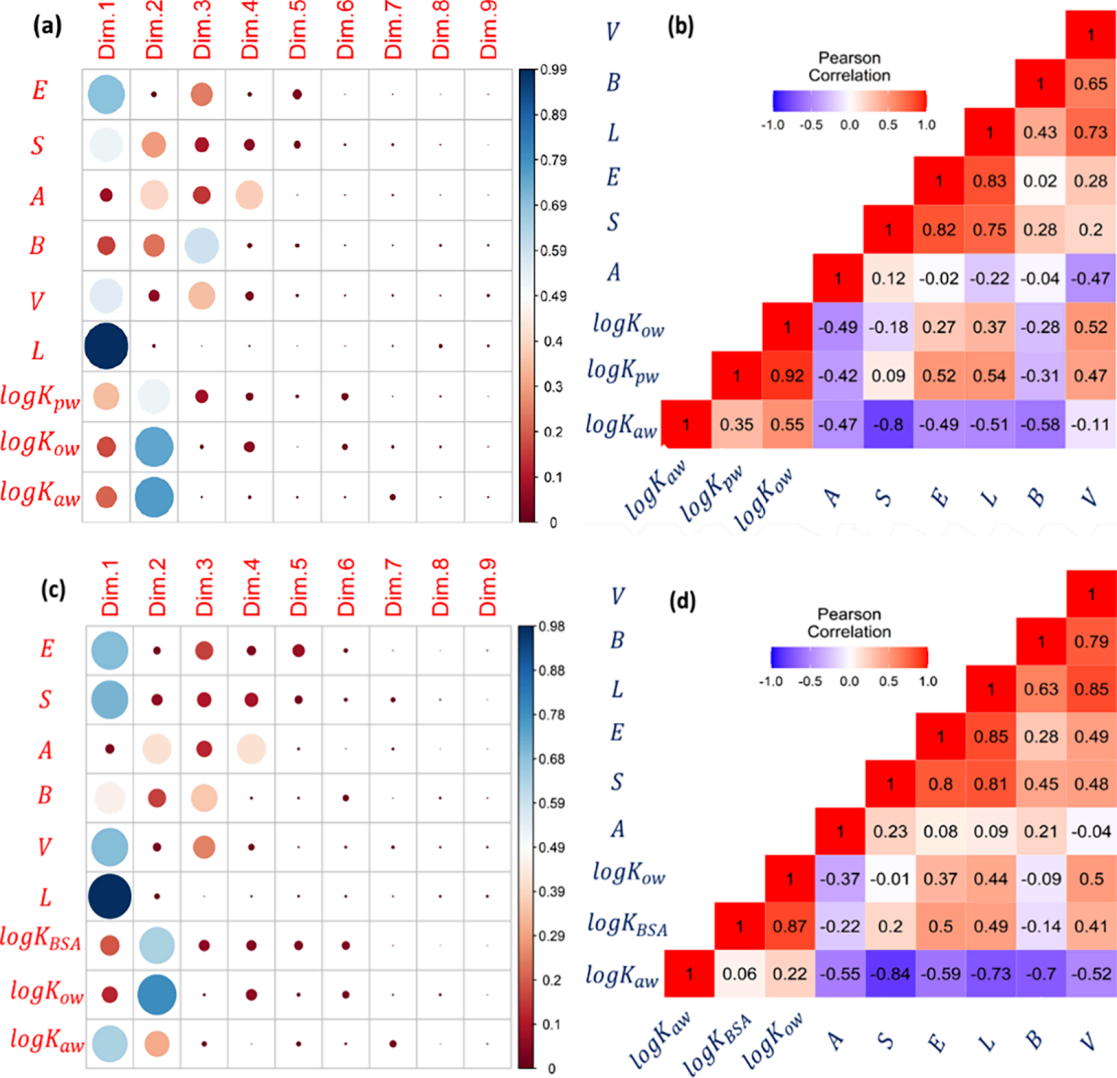
Dimensionality analyses on the calibration data sets for *2p*-LFER models of *logK*_*PW*_ and *logK*_*BSA*_. The upper panels show the results obtained by (a) the Principal Component Analysis (PCA) and (*b*) Pearson Correlation Analysis performed on 51 × 9 matrix, [*logK*_*pw*_, *E*, *S*, *A*, *B*, *V*, *L, logK*_*ow*_, *logK*_*aw*_]. The lower panels show the results of (**c**) PCA and (**d**) Pearson Correlation Analysis on 83 × 9 matrix, [*logK*_*BSA*_, *E*, *S*, *A*, *B*, *V*, *L*, *logK*_*ow*_*, logK*_*aw*_].

As demonstrated in Figures 1 and 2, log *K*_aw_ predominantly captures the hydrogen bonding interactions, more so than log *K*_ow_. This indicates that hydrogen bonding interactions play a more significant role in the partitioning behavior of proteins compared to lipids. This distinction highlights the differential importance of hydrogen bonding in the partitioning processes of proteins versus lipid-based phases. This can be further corroborated by looking at the solvation characteristics of storage lipid–water,^36^ phospholipid–water,^37^ and structural protein–water^14^ phases, which are distinctively outlined by Abraham’*s* model parameters. The storage lipid–water phase displays pronounced hydrophobicity, as indicated by the highest *l* coefficient, and the most negative *s*, *a*, and *b* values. This suggests a strong affinity for nonpolar interactions. Conversely, the structural protein–water phase emerges as least hydrophobic and more accommodating to hydrogen bonding and polar chemicals evidenced by the least *a*, *b* and *s* coefficient. The phospholipid–water phase presents intermediate properties, balancing between hydrophobicity and polarity. Consequently, storage lipids are inferred to preferentially partition more hydrophobic and nonpolar compounds, while structural proteins are more receptive to hydrogen bonding, highlighting the diverse solvation dynamics within biological systems.

### 3.4 Bovine Serum Albumin

The linear regression analysis revealed that both log *K*_ow_ and log *K*_aw_ are significant predictors of log *K*_BSA_, with log *K*_ow_ having a more pronounced effect (Table 1). This model explained approximately 76% of the variance in log *K*_BSA_, indicating a significant relationship between these partitioning behaviors. The positive coefficient for log *K*_ow_ suggests that as the affinity of a compound for octanol over water increases, so does its affinity for binding to bovine serum albumin. In contrast, the negative coefficient for log *K*_aw_ suggests an inverse relationship for air–water partitioning. The presence of significant coefficients for both log *K*_ow_ and log *K*_aw_ in predicting log *K*_BSA_ underscores the complex interplay between different types of partitioning behaviors in biological systems. The positive relationship with log *K*_ow_ aligns with the understanding that compounds with higher lipophilicity (as indicated by a higher octanol–water partition coefficient) tend to have higher affinity for albumin binding. The inverse relationship between log *K*_BSA_ and log *K*_aw_ may be attributed to the polarity and hydrogen bonding interactions of chemicals, with log *K*_aw_ serving as a proxy (Figures 1 and 2). This suggests a preferential transfer of chemicals from the albumin to the water phase due to these interactions. These results are valuable for understanding and predicting how different compounds might behave in biological systems, particularly in relation to their distribution and binding characteristics.

Further analysis of the model reveals significant differences in the impact of dropping either log *K*_ow_ or log *K*_aw_ on the performance of the model. Removing log *K*_ow_ leads to a poorly performing model, as evidenced by a negative *R*^2^ value and a substantially higher RMSE. This indicates that log *K*_ow_ is a crucial predictor for log *K*_BSA_, significantly contributing to the accuracy and explanatory power of the model. Conversely, omitting log *K*_aw_ results in a moderate decline in the model’*s* performance, with a decrease in *R*^2^ and an increase in RMSE, but not to the extent observed with the removal of log *K*_ow_. This suggests that while log *K*_aw_ has a role in the model, its influence is less pronounced compared to log *K*_ow_. Therefore, log *K*_ow_ is a more critical variable in predicting the partition coefficient between bovine serum albumin and water (log *K*_BSA_).

The cross-validation results for the linear regression model predicting the log *K*_BSA_ from log *K*_ow_ and log *K*_aw_ provide an evaluation of the robustness and predictive power of the model. The hold-out method, with a training-to-testing ratio of 1:4 (Tables S13 and S14), showed a high *R*^2^ value of 0.847 and an RMSE of 0.338, indicating strong predictive performance on the test set (eq 2). However, reliance on a single train-test split might not fully capture the generalizability model.
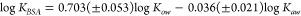
2

The comparison of the regression coefficients between the main model (eq 1) and the hold-out model (eq 2) reveals that the differences in coefficients for log *K*_ow_ and log *K*_aw_ are not statistically significant. The calculated z-scores for both coefficients fall below the threshold of 1.96, typically used to denote significance at the 5% level. This finding indicates that the observed variations in coefficients between the two models are likely due to sampling variability and do not reflect substantial differences in the underlying relationships between the variables. Therefore, despite the slight numerical differences in coefficients, the models are statistically consistent with each other in terms of the effects of log *K*_ow_ and log *K*_aw_ on log *K*_BSA_.

The *k*-fold and repeated *k*-fold cross-validation methods, which mitigate the potential overfitting or underfitting issues of the hold-out method by averaging results over multiple splits, showed mean *R*^2^ values of 0.690 and 0.709, respectively. These values, along with their associated standard deviations (0.195 for *k*-fold and 0.134 for Repeated *k*-fold), suggest that while the model performs well on average, there is variability in its performance across different subsets of the data. The bootstrap method, with 1000 iterations and a 50% sample size, provided a stable mean *R*^2^ of 0.746 with a low standard deviation of 0.021, indicating consistent model performance across various resampled data sets. Overall, these cross-validation results underscore the model’*s* reliability in predicting log *K*_BSA_, with certain variability depending on the cross-validation method used. This highlights the importance of using diverse validation techniques to assess a model’*s* performance comprehensively, especially in cases where data may have unique properties or when working with smaller data sets.

In assessing the partitioning behaviors of chemicals with log *K*_BSA_ and log *K*_pw_, hydrophobicity (log *K*_ow_) positively influences both, albeit more so for structural proteins, indicating a greater sensitivity to hydrophobic interactions. Conversely, the proxy for volatility/solubility (log *K*_aw_) negatively impacts partitioning with both proteins, with structural protein showing a stronger negative response. This suggests that structural protein’*s* interaction with chemicals is more sensitive to both the hydrophobicity and volatility/solubility traits compared to bovine serum albumin. These trends highlight the nuanced differences in how these proteins interact with chemicals, emphasizing the complexity of protein-chemical interactions influenced by multiple physicochemical properties.

Comparing the partition coefficient between bovine serum albumin and water (log *K*_BSA_) with those of storage lipid (log *K*_*lw*_) and phospholipids (log *K*_*phw*_) reveals distinct interaction patterns based on hydrophobicity (log *K*_ow_) and the volatility/solubility (log *K*_aw_). All three models show a positive relationship with log *K*_ow_, indicating that more hydrophobic compounds have higher affinity across these biological matrices. However, this hydrophobic interaction is more pronounced in the lipid models, with storage lipids and phospholipids exhibiting higher coefficients than bovine serum albumin. In terms of log *K*_aw_, bovine serum albumin and phospholipids display a negative relationship, suggesting a decreased affinity for more volatile compounds. Conversely, storage lipids show a positive correlation with log *K*_aw_, implying a different interaction mechanism, likely influenced by their role in storing substances. These contrasts highlight the varied and complex nature of chemical interactions with different biological components, with lipids showing a stronger hydrophobic influence and varying responses to compound volatility compared to albumin.

The Abraham solvation parameters offer a comparative view of the partitioning behaviors for chemicals within four distinct systems: structural protein–water,^14^ bovine serum albumin-water,^15^ storage lipid–water,^36^ and phospholipid–water.^37^ The polarity/polarizability (*s*) and hydrogen bond acidity (*b*) parameters are negative for all, suggesting a universal trend where polar and hydrogen bond-donating chemicals favor the aqueous phase. However, the more negative *s* and *b* for storage lipid–water reflect its particularly low affinity for these interactions, likely due to its hydrophobic nature. Conversely, the least negative *s* and *b* for bovine serum albumin indicate a relatively higher tolerance for polarity and hydrogen bond donation within the protein phase.

Hydrogen bond basicity (*a*) follows a similar trend, with negative values for storage lipid and phospholipid–water phases, highlighting water’*s* dominance in accepting hydrogen bonds. In contrast, the positive *a* for bovine serum albumin suggests a unique capability among the biological phases to accommodate hydrogen bond acceptors, consistent with the diverse functionality of serum albumins.

The McGowan volume (*v*) coefficients are positive across all systems, indicating a general preference for larger solutes in the biological phases. The magnitude of *v* varies, with structural protein–water showing the highest value, implying a greater propensity to accommodate bulkier solutes, perhaps due to the intricate tertiary structure of proteins providing more spatial accommodation.

In summary, while all four systems exhibit a tendency to partition polar and hydrogen-bonding solutes toward water, the degree of this preference is most pronounced in storage lipids. Bovine serum albumin stands out for its ability to interact with hydrogen bond acceptors, and structural protein’*s* capacity for larger solutes is notable. These distinctions underscore the unique solvation characteristics inherent to each biological phase, revealing the complexity of solute interactions within biologically relevant environments.

### 3.5 Assessing *2p*-LFER Predictive Accuracy for Per- and Polyfluoroalkyl Substances

Per- and polyfluoroalkyl substances (PFAS), commonly known as forever chemicals, pose significant environmental health concerns.^38^ The effectiveness of the *2p*-LFER models was evaluated for two distinct sets of PFAS. Notably, PFAS were not included in the original chemical data sets used to train the *2p*-LFER models for log *K*_pw_ and log *K*_BSA_.

**Figure 3:**
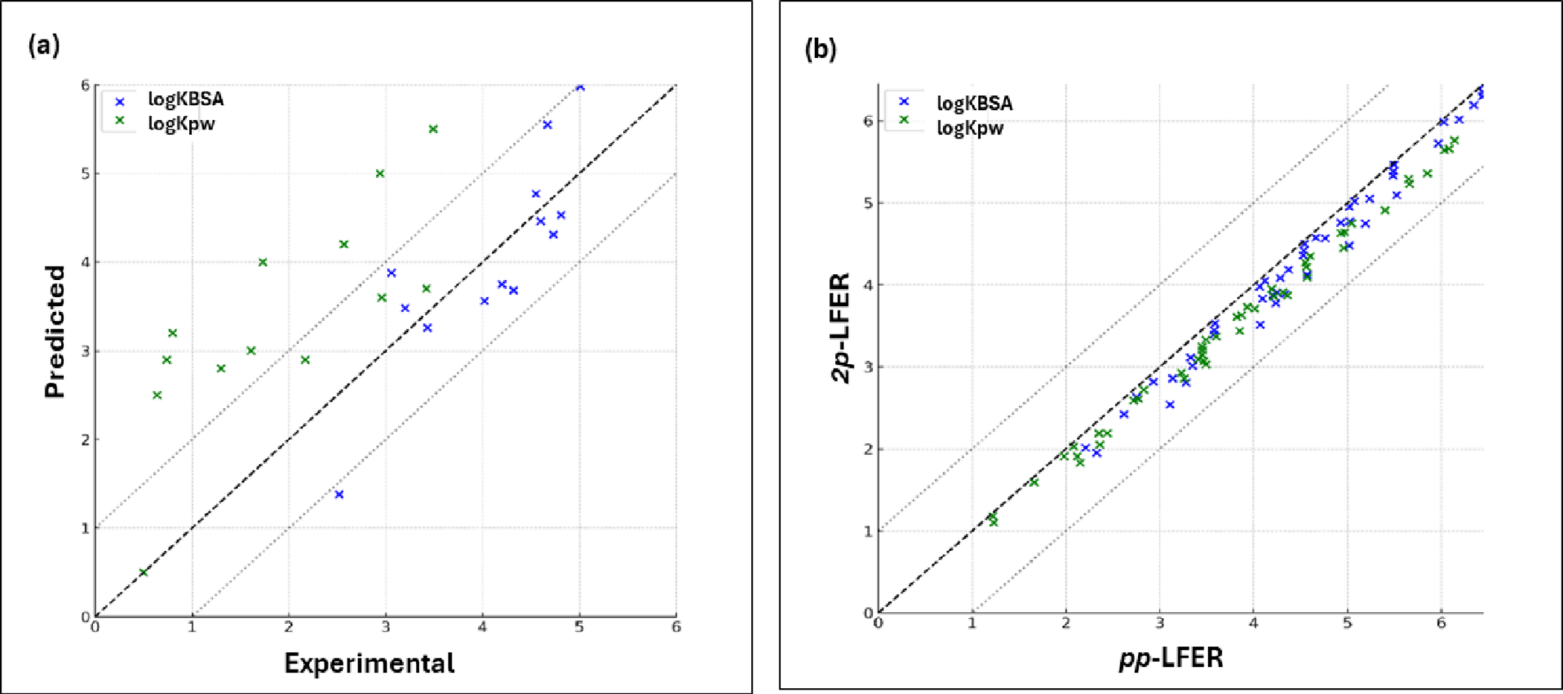
Comparison of predicted and experimental/reference partition coefficients for bovine serum albumin to water (log *K*_BSA_) and structural protein to water (log *K*_pw_) systems. Panel (a) displays the predicted values against experimental data for 13 ionizable perfluoroalkyl acids and sulfonates. Panel (b) contrasts the predicted values from *2p*-LFERs with reference values obtained via *pp*-LFERs for 47 neutral fluorotelomer compounds, encompassing subcategories such as fluorotelomer alcohols, iodides, olefins, acrylates, and methacrylates.

The first evaluated group consisted of 13 ionizable perfluoroalkyl acids and sulfonates (Table S7). For these chemicals, experimental values of log *K*_pw_ and log *K*_BSA_ were available in the literature, providing a basis for direct comparison with the *2p*-LFER model predictions. In this comparison, the *2p*-LFER model’s predictions for log *K*_pw_ across 12 substances showed a RMSE of 1.71 log units, indicating a significant deviation from the experimental values. Conversely, the predictions for log *K*_BSA_ across 13 substances were more accurate, with an RMSE of 0.61 log units (Figure 3a).

The second group of chemicals assessed comprised 47 neutral fluorotelomer compounds, including various subcategories like fluorotelomer alcohols, iodides, olefins, acrylates, and methacrylates (Table S8). For these compounds, lacking experimental log *K*_pw_ and log *K*_BSA_ values, estimates were made using their Abraham solute descriptors. When these estimates were compared with the *2p*-LFER predictions, the results were more encouraging (Figure 3b). The RMSE for log *K*_pw_ predictions was 0.30 log units, and for log *K*_BSA_, it was 0.27 log units. These lower RMSE values indicate a closer match between the predicted and estimated values, suggesting that the *2p*-LFER model performs well for neutral fluorotelomer compounds. This finding supports the model’s suitability for a certain range of PFAS, particularly those that are neutral and less complex in nature.

In summary, while the *2p*-LFER model shows promising results for certain classes of PFAS, particularly neutral fluorotelomer compounds, its applicability is limited for ionizable PFAS due to the overestimation of log *K*_pw_ values. These findings highlight the need for model refinement or the development of specialized models to accurately predict the environmental behavior of a broader range of PFAS, especially those with ionizable properties. Given these insights, the scope for advancing our model’s predictive capacity through feature engineering could be an interesting future research direction. Although feature engineering techniques may not traditionally align with the foundational principles of LFERs—valued for their simplicity and interpretability—embracing such approaches could unlock new avenues for accurately modeling PFAS behavior. By venturing beyond the conventional domain of LFERs, future studies could explore the integration of complex, nonlinear descriptors or features, tailored to capture the unique properties of ionizable PFAS.

Next, we aimed to investigate the extent to which proteins contribute to bioaccumulation compared to lipids, which are typically used to estimate bioaccumulation. To achieve this, we calculated the partition coefficients—— log *K*_pw_, log *K*_BSA_, log *K*_*lw*_, and log *K*_*phw*_ —for the PFAS in the second group using *2p*-LFERs developed in both our current and prior studies. The relative distribution of PFAS across storage lipids, phospholipids (membrane lipids), serum proteins (albumin), and structural proteins in various organs was determined by applying their respective partition coefficients and considering the relative fractions of these phases within the organs’ tissues, assuming multiphase equilibrium partitioning.

**Figure 4:**
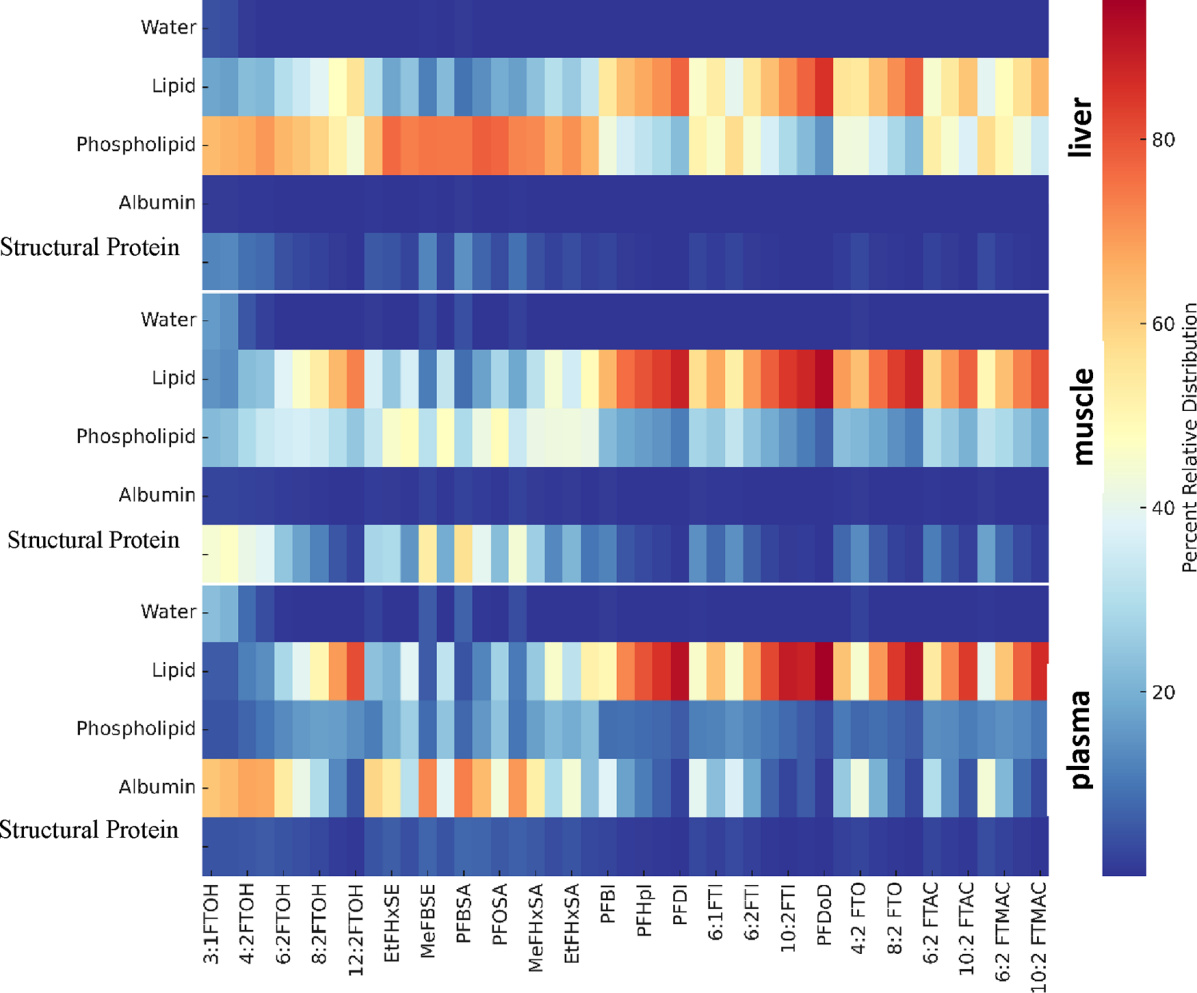
Relative distributions of neutral per- and polyfluoroalkyl substances (PFAS) across various phases including water, albumin, structural proteins, lipids, and phospholipids in mammalian tissues/fluids such as plasma, muscle, and liver. These distributions are obtained through multiphase partitioning modeling based on predicted partition coefficients via *2p*-LFER.

Our analysis revealed that in protein-enriched tissues such as the liver, muscle, and plasma, the relative contribution of PFAS load in structural and plasma proteins is significant, and in several cases, it is even equal to or higher than that of lipids (Figure 4). Similar results were obtained when multiphase partitioning model was parametrized with the partition coefficients estimated via *pp*-LFERs (Figure S2). This suggests that the partitioning of PFAS to these proteins should be taken into account as an important factor when evaluating the bioaccumulation of these chemicals.

### 3.6 Indirect Validation of *2p*-LFERs through Multiphase Equilibrium Partitioning Models

**Figure 5:**
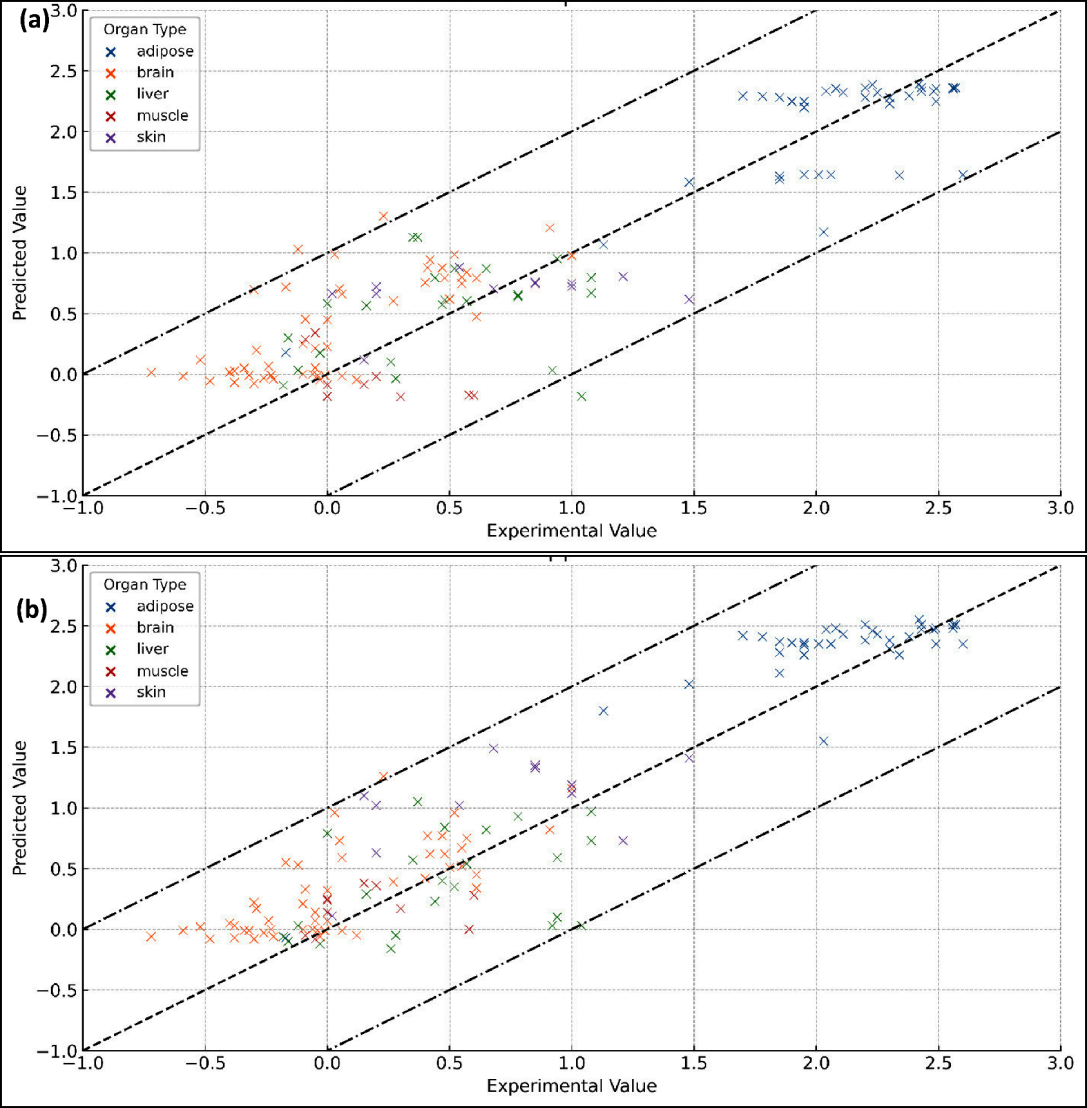
Experimental *in vivo* and *in vitro* distribution ratios for various mammalian organs compared to their predicted values obtained through multiphase equilibrium partitioning model. This model is parametrized with the input of (a) *2p*-LFER and (b) *pp*-LFER predicted partition coefficients for lipids and proteins.

Our analysis of the multiphase partitioning model, parametrized with *2p*-LFER-derived coefficients, yielded *in vivo* and *in vitro* distribution data that closely aligned with experimental observations (Figure 5a). The model’s predictions demonstrated a RMSE of 0.44 log units, attesting to the model’s precision. Notably, a parallel model, employing *pp*-LFERs as a parametrization foundation, attained a marginally lower RMSE of 0.42 log units (Figure 5b). When comparing the predicted and experimental milk-water partitioning data (n = 108, Table S10), the multiphase partitioning model, parametrized with *2p*-LFER-derived partition coefficients, showed good agreement, with an RMSE of 0.29 log units (Figure S3). In contrast, the model parametrized on *pp*-LFER estimated partition coefficient yielded a marginally improved RMSE of 0.25 log units against the same data set.

Notably, the multiphase model parametrized with estimated partition coefficients for lipid and proteins using historical one-parameter-based LFERs (*1p*-LFERs) — which are based on log *K*_ow_ — yielded predictions with an RMSE of 0.59 log units when compared to the same experimental data set. This result aligns with our current and previous observations that *1p*-LFERs are not as accurate as *2p*- and *pp*-LFERs. This comparative exercise underscores the comparable efficacy of *2p*-LFERs with *pp*-LFERs, suggesting that *2p*-LFERs can be reliably utilized in scenarios where *pp*-LFERs may not be suitable, particularly in the absence of adequate ASDs. These findings substantiate the applicability of *2p*-LFERs in predictive partitioning models and reinforce their potential as a complementary tool in chemical distribution studies.

## 4 Integration Assessment of *2p*-LFER Models for EPI Suite

The Estimation Program Interface Suite (EPI Suite) by the US EPA and Syracuse Research Corp. provides valuable predictions on environmental properties, fate, and ecotoxicity of chemicals. However, it currently lacks a module to predict log *K*_pw_ and log *K*_BSA_. An evaluation for integrating *2p*-LFER models into EPI Suite involved using estimated log *K*_ow_ and log *K*_aw_ values from EPI Suite as inputs to the *2p*-LFER equations for log *K*_pw_ and log *K*_BSA_. These models showed promising results, with RMSE of 0.37 and 0.50, respectively, closely aligning with experimental data. The robustness of estimated log *K*_ow_ and log *K*_aw_ as input parameters was also confirmed, with low RMSEs when compared to their experimental counterparts. This analysis suggests that the *2p*-LFER models could be effectively integrated into EPI Suite, enhancing its capability to reliably predict log *K*_pw_ and log *K*_BSA_ coefficients.

Our preceding research^22^ rigorously assessed the accuracy of log *K*_ow_ and log *K*_aw_ values derived from EPI Suite. This assessment revealed that EPI Suite’s performance in predicting log *K*_ow_ and log *K*_aw_ closely matched that of the ASM, yielding RMSEs of 0.28 and 0.26 for log *K*_ow_, and 0.50 for log *K*_aw_, against experimental values from 304 and 296 compounds, respectively. This level of accuracy affirms the reliability of EPI Suite-sourced parameters for our *2p*-LFER models.

However, caution is warranted for PFAS, given recent findings^29^ that highlight discrepancies in EPI Suite’s predictions of log *K*_aw_ when compared to advanced quantum chemical models like COSMOtherm, despite ASM showing good agreement. The absence of experimental data for direct comparison restricts this evaluation to predictions from different models. These findings underscore the potential limitations of using EPI Suite-estimated parameters for PFAS within our 2p-LFER framework, suggesting careful parameter selection is essential, particularly for chemicals with complex or unique attributes.

## 5 Application Domain and Limitations of *2p*-LFER Models

In the structural protein–water partitioning model, the majority of observations align with the applicability domain, highlighting the efficacy of model in predicting the partitioning behavior of structural proteins using log *K*_ow_ and log *K*_aw_ as independent variables. However, deviations were observed in four specific chemicals: 1-chlorooctane, tri-*n*-butyl phosphate, 4-ethyl-3-hexanol, and benzo[a]pyrene. These deviations manifested either as high leverage, indicating a substantial influence of their log *K*_ow_ and log *K*_aw_ values, or as discrepancies in standardized residuals, reflecting differences between predicted and observed log *K*_pw_ values. Notably, benzo[a]pyrene (log *K*_ow_: 6.13, log *K*_aw_: −4.73, log *K*_pw_: 4.925), 1-chlorooctane (log *K*_ow_: 3.64, log *K*_aw_: 0.19, log *K*_pw_: 2.905), tri-*n*-butyl phosphate (log *K*_ow_: 4.00, log *K*_aw_: −4.24, log *K*_pw_: 1.760), and 4-ethyl-3-hexanol (log *K*_ow_: 2.78, log *K*_aw_: −2.85, log *K*_pw_: 0.750) exhibited either extreme hydrophobicity or volatility/solubility, which could impede accurate measurements of their properties. These attributes potentially render them incompatible within the typical range of data set. Consequently, it is challenging to determine whether the observed deviations are due to limitations of the model or inaccuracies in the experimental data for these compounds with extreme properties. Nevertheless, excluding these outliers from the training data set did not alter the fitting coefficients of model, indicating the robustness of the model’s core structure despite the presence of these anomalous observations.

**Figure 6:**
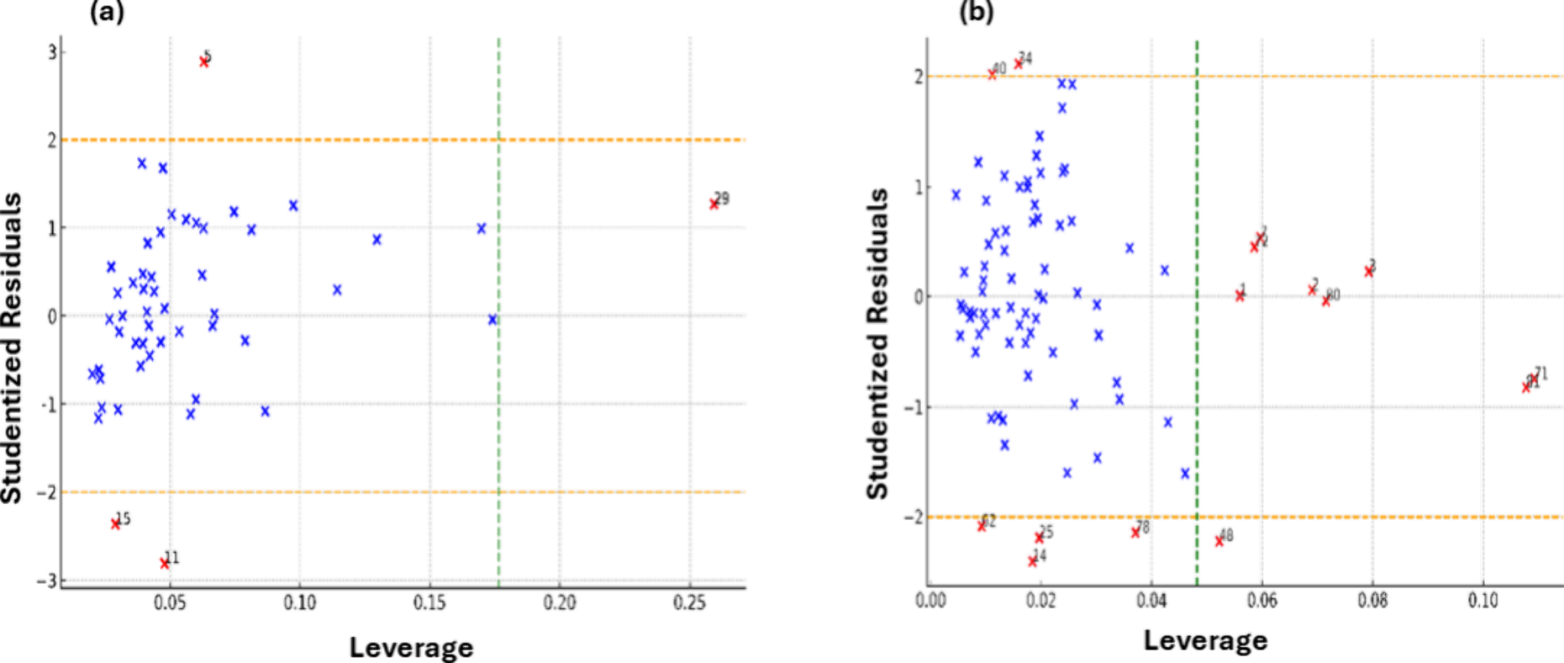
William’s Plot highlighting influential observations in the data sets for (a) structural protein and (b) bovine serum albumin. Observations with standardized residuals beyond ±2 or leverage higher than 0.06 are marked in red and annotated with their index numbers, indicating potential outliers or influential points for the model. The orange horizontal lines representing the ±2 standardized residual threshold and the green vertical line indicating the leverage threshold.

Several chemicals in the data set fall comfortably within the applicability domain of our linear regression model, which successfully predicts the log *K*_BSA_ (Figure 6). This indicates the model’*s* robustness and reliability for a diverse range of compounds. However, the William’*s* plot analysis has also highlighted a few chemicals that show deviations from the model’*s* predictions. These influential chemicals, such as *n*-heptane, *n*-octane, γ-hexachlorocyclohexane, and diazepam, exhibit a wide range of values in their partition coefficients in octanol–water and air–water systems. Some, like bisphenol A and estrone, have very negative log *K*_aw_ values, indicating a markedly low volatility, whereas others like *n*-octane and *n*-nonane demonstrate a strong affinity for octanol as suggested by their high log *K*_ow_ values. Accurate measurement of partition coefficients for such extreme cases is challenging. This makes it difficult to ascertain whether the model is unsuitable for these compounds or if the discrepancies arise from data quality issues. Addressing this uncertainty represents an interesting direction for future research. The identification of these outliers is crucial for understanding the limits of the model’*s* applicability and for ensuring accurate interpretations, particularly for compounds with extreme partitioning behaviors. These findings underscore the importance of considering a model’*s* domain of applicability and the influence of individual observations on its performance.

An inherent limitation of LFER models, including our 2p-LFER approach, lies in their design primarily for neutral chemicals. Predicting behaviors for ionized species necessitates integrating specific descriptors, such as those based on dissociation constants (e.g., p*K*_a_ values), as well as metrics that capture ionic interactions and fluorine-specific characteristics (e.g., electronegativity or fluorination patterns), to precisely account for ionization states and environmental interactions.^39^ The inclusion of such descriptors for PFAS—a class of chemicals lacking extensive ionization and partitioning data—remains beyond the scope of this study due to data availability constraints. Future work could address these limitations by advancing models with descriptors tailored to PFAS-specific properties, offering improved accuracy for this distinctive class of compounds.

## 6 Conclusions

In conclusion, our study demonstrates that *2p*-LFERs effectively capture the variance in structural proteins and bovine serum albumin data. The estimates of partition coefficients for structural proteins, bovine serum albumin, storage lipid, and phospholipid, as derived from *2p*-LFERs, show promise for use in multiphase partitioning models. These models, when combined with the tissue composition data of these phases within organs, can predict the *in vivo* and *in vitro* distribution of a diverse range of organic chemicals effectively.

However, it is crucial to approach the use of *2p*-LFERs with caution, especially for chemicals at the extreme ends of hydrophobicity and volatility spectra, as well as for ionizable compounds. The accuracy of log *K*_ow_ and log *K*_aw_ values for such chemicals can be compromised, which in turn affects the reliability of *2p*-LFER predictions based on these inputs. While *2p*-LFERs have shown questionable performance for ionizable PFAS, their effectiveness is notable for neutral PFAS.

Employing *2p*-LFERs could potentially offer valuable insights across environmental and toxicological modeling, suggesting essential improvements in current practices. The conventional octanol-based *1p*-LFERs have their utility in screening scenarios, such as multimedia fate modeling, where an estimation error within 1 order of magnitude is acceptable for biosorption. However, for more precise estimates of bioaccumulation, internal concentrations, and organ-specific toxicity, *pp*-LFER based multiphase partitioning models prove to be more suitable.^12^ Nonetheless, it is important to note that *pp*-LFER descriptors are limited to approximately 8000 chemicals. In instances where *pp*-LFER descriptors are unavailable, our two-parameter LFERs (*2p*-LFERs) offer comparably accurate estimates of sorptive capacities of various organs using multiphase partitioning approach. This accuracy is particularly relevant for chemicals that engage in hydrogen bonding interactions or exhibit hydrophilic characteristics, as the *1p*-LFERs tend to be less reliable than those from *2p*- and *pp*-LFERs. Similarly, for calculating benchmarks such as biomagnification factor (BMF) and trophic magnification factor (TMF) — traditionally derived from octanol-based *1p*-LFER — the resulting fugacity capacities can exhibit errors greater than one log unit.^12^ Such inaccuracies are unsuitable for regulatory purposes, which demand more precise estimations. In these contexts, our *2p*-LFER model emerges as a viable alternative to ASMs, offering enhanced accuracy and reliability for environmental assessments.

Overall, *2p*-LFERs present themselves as valuable models, especially in cases where *pp*-LFERs are limited by the absence of experimental Abraham solute descriptors. This study thus contributes to the broader field by offering an alternative modeling approach, while also highlighting areas for cautious application and further research.

## Data Availability

Data, including all molecular structures and their properties, are available in a machine-readable format as Supporting Information and on Zenodo (https://doi.org/10.5281/zenodo.12624094).

## Supporting Information Available

Detailed tables (Tables S1–S14) covering chemicals, their experimental values, and calibration models for various protein–water partition coefficients; figures (Figures S1–S4) illustrating the comparison of partition coefficients, distribution of PFAS substances, and PCA plots on Abraham solute descriptors; and the R codes used to generate figures and results (Section 2) (PDF)
