# Supplementary material for: Streamlining Linear Free Energy Relationships of Proteins through Dimensionality Analysis and Linear Modeling
Source: J Chem Inf Model. 2024 Dec 3;64(24):9327–40. doi: 10.1021/acs.jcim.4c01289 (PMC11683862; doi:10.1021/acs.jcim.4c01289)
Supplement: Supplementary file 1 — ci4c01289_si_001.pdf [file ci4c01289_si_001.pdf]

Supporting Information

Streamlining Linear Free Energy Relationships of Proteins through Dimensionality Analysis and Linear Modeling

Muhammad Irfan Khawar[1], Muhammad Arshad[1], Eric P. Achterberg[2], Deedar Nabi[1,2*]

[1]Institute of Environmental Sciences and Engineering, School of Civil and Environmental Engineering, National University of Sciences and Technology (NUST), H-12, Islamabad, Pakistan.

[2]GEOMAR Helmholtz Centre for Ocean Research Kiel, Wischhofstr. 1-3, 24148 Kiel, Germany

*Corresponding author
Email: dnabi@geomar.de

Table S 1: Chemicals with their experimental values of $logKch$, and values of $logKow$ and $logKaw$ used to calibrate chicken muscle protein-water 2p-LFER model. 3
Table S 2: Chemicals with their experimental values of $logKfish$ and values of $logKow$ and $logKaw$ used to calibrate fish muscle protein-water 2p-LFER model. 5
Table S 3: Chemicals with their experimental values of $logKBSA$ and values of $logKow$ and $logKaw$ used to calibrate bovine serum albumin-water 2p-LFER model. 8
Table S 4: Chemicals with their experimental values of $logKpw$ and values of $logKow$ and $logKaw$ used to calibrate combined chicken and fish muscle protein-water 2p-LFER model. 14
Table S 5: Diversity of data for $logKpw$....17
Table S 6: Diversity of data for $logKBSA$....17
Table S 7: Comparison of Experimental and 2p-LFER Predicted Partition Coefficients for ionizable PFAS Compounds....18
Table S 8: List of neutral fluorotelomer PFAS Compounds....19
Table S 9: List of experimental in vivo and in vitro partitioning data for different tissues and species. 21
Table S 10: List of experimental Milk-water partition coefficient and predicted values of Milk-water partitioning. 26
Table S 11: Training set for $logKpw$ 31
Table S 12: Validation set for $log\ Kpw$ 33
Table S 13: Training set for $log\ KBSA$ 34
Table S 14: Validation set for $log\ KBSA$ 39

Figure S 1 Bland-Altman Plot illustrating the agreement between fish and chicken protein-water partition coefficients. 41
Figure S 2: Relative distribution of neutral per- and polyfluoroalkyl substances (PFAS) across various phases 42

Figure S 3: The agreement between experimental milk-to-water partition coefficients and predicted values using 2p-LFER. 43
Figure S 4:. PCA plots performed on Abraham solute descriptors (ASDs) of $logKch$ (a), $logKfish$(b), $logKpw$ (c), and $logKBSA$ (d) indicating the values of first two dimensions. 44

Section 1: Cross validation of all models 45
Section 2. R codes used to analyze 46
References 51

Table S 1: Chemicals with their experimental values of $logK_{ch}$, and values of $logK_{ow}$ and $logK_{aw}$ used to calibrate chicken muscle protein-water 2p-LFER model.[a]

S. No	Chemicals	CAS-RN	SMILES	$logK_{ch}$	$logK_{ow}$	$logK_{aw}$
1	*n-Nonane*	*111-84-2*	*CCCCCCCCC*	*3.86*	*5.65*	*2.14*
2	*Cyclooctane*	*292-64-8*	*C1CCCCCCC1*	*2.94*	*4.45*	*1.18*
3	*Cycloheptane*	*291-64-5*	*C1CCCCCC1*	*2.16*	*4.00*	*0.58*
4	*Non-1-ene*	*124-11-8*	*CCCCCCCC=C*	*3.54*	*5.15*	*1.51*
5	*Hept-1-yne*	*628-71-7*	*CCCCCC#C*	*1.75*	*3.44*	*0.46*
6	*1-Chlorooctane*	*111-85-3*	*CCCCCCCCCl*	*2.93*	*3.64*	*0.19*
7	*Di-n-butyl ether*	*142-96-1*	*CCCCOCCCC*	*1.72*	*3.21*	*-0.61*
8	*Octan-2-one*	*111-13-7*	*CCCCCCC(=O)C*	*1.57*	*2.37*	*-2.11*
9	*Nonan-2-one*	*821-55-6*	*CCCCCCCC(=O)C*	*1.83*	*3.14*	*-1.82*
10	*Decan-2-one*	*693-54-9*	*CCCCCCCCC(=O)C*	*2.11*	*3.73*	*-1.91*
11	*1-Nitrohexane*	*646-14-0*	*CCCCCCN(=O)=O*	*1.34*	*2.70*	*-2.03*
12	*Tri-n-butyl phosphate*	*126-73-8*	*CCCCOP(=O)(OCCCC)OCCCC*	*1.69*	*4.00*	*-4.24*
13	*Octan-1-ol*	*111-87-5*	*CCCCCCCCO*	*1.42*	*3.00*	*-3.00*
14	*Nonan-1-ol*	*143-08-8*	*CCCCCCCCCO*	*1.97*	*3.77*	*-2.90*
15	*n-Propylbenzene*	*103-65-1*	*CCCc1ccccc1*	*1.98*	*3.69*	*-0.37*

S. No	Chemicals	CAS-RN	SMILES	$logK_{ch}$	$logK_{ow}$	$logK_{aw}$
16	*n-Pentylbenzene*	*538-68-1*	*CCCCCc1ccccc1*	*2.71*	*4.90*	*0.02*
17	*n-Hexylbenzene*	*1077-16-3*	*CCCCCCc1ccccc1*	*3.19*	*5.52*	*0.07*
18	*1,2,4-Trimethylbenzene*	*95-63-6*	*Cc1ccc(c(c1)C)C*	*1.9*	*3.63*	*-0.60*
19	*1,2-Dichlorobenzene*	*95-50-1*	*Clc1ccccc1Cl*	*2.02*	*3.43*	*-1.11*
20	*1,2,3,4-Tetrachlorobenzene*	*634-66-2*	*Clc1c(Cl)ccc(c1Cl)Cl*	*2.8*	*4.60*	*-1.51*
21	*1,2,4,5-Tetrachlorobenzene*	*95-94-3*	*Clc1cc(Cl)c(cc1Cl)Cl*	*2.94*	*4.64*	*-1.39*
22	*Pentachlorobenzene*	*608-93-5*	*Clc1cc(Cl)c(c(c1Cl)Cl)Cl*	*3.35*	*5.17*	*-1.54*
23	*1,4-Dibromobenzene*	*106-37-6*	*Brc1ccc(cc1)Br*	*2.18*	*3.79*	*-1.44*
24	*Dibenzothiophene*	*132-65-0*	*c1ccc2c(c1)sc1c2cccc1*	*3.4*	*4.38*	*-2.86*
25	*Phenanthrene*	*85-01-8*	*c1ccc2c(c1)c1ccccc1cc2*	*3.14*	*4.46*	*-2.76*
26	*Pyrene*	*129-00-0*	*c1cc2ccc3c4c2c(c1)ccc4ccc3*	*3.67*	*4.88*	*-3.31*
27	*Chrysene*	*218-01-9*	*c1ccc2c(c1)c1ccc3c(c1cc2)cccc3*	*4.53*	*5.81*	*-3.67*
28	*Benzo[a]pyrene*	*50-32-8*	*c1ccc2c(c1)c1ccc3c4c1c(c2)ccc4ccc3*	*4.88*	*6.13*	*-4.73*
29	*4-Nitroanisole*	*100-17-4*	*COc1ccc(cc1)N(=O)=O*	*1.38*	*2.03*	*-4.21*
30	*Valerophenone*	*1009-14-9*	*CCCCC(=O)c1ccccc1*	*1.74*	*3.29*	*-2.98*
31	*Benzophenone*	*119-61-9*	*O=C(c1ccccc1)c1ccccc1*	*2.01*	*3.18*	*-4.46*
32	*2-Nitrotoluene*	*88-72-2*	*O=N(=O)c1ccccc1C*	*1.27*	*2.30*	*-3.29*
33	*2,4-Dinitrotoluene*	*121-14-2*	*O=N(=O)c1ccc(c(c1)N(=O)=O)C*	*1.22*	*1.98*	*-5.66*

S. No	Chemicals	CAS-RN	SMILES	$logK_{ch}$	$logK_{ow}$	$logK_{aw}$
34	*4-n-Propylphenol*	*645-56-7*	*CCCc1ccc(cc1)O*	*1.69*	*3.20*	*-4.33*
35	*4-Fluorophenol*	*371-41-5*	*Oc1ccc(cc1)F*	*1.18*	*1.77*	*-4.54*
36	*3-Chlorophenol*	*108-43-0*	*Oc1cccc(c1)Cl*	*1.57*	*2.50*	*-4.85*
37	*4-Chlorophenol*	*106-48-9*	*Oc1ccc(cc1)Cl*	*1.55*	*2.39*	*-4.59*
38	*4-Bromophenol*	*106-41-2*	*Oc1ccc(cc1)Br*	*1.7*	*2.59*	*-5.21*
39	*4-Iodophenol*	*540-38-5*	*Oc1ccc(cc1)I*	*1.93*	*2.91*	*-5.41*
40	*2-Phenylphenol*	*90-43-7*	*Oc1ccccc1c1ccccc1*	*2*	*3.09*	*-4.37*
41	*4-Chloroaniline*	*106-47-8*	*Nc1ccc(cc1)Cl*	*1.19*	*1.83*	*-4.32*
42	*4-Iodoaniline*	*540-37-4*	*Nc1ccc(cc1)I*	*1.66*	*2.34*	*-5.14*
43	*4-Nitroaniline*	*100-01-6*	*Nc1ccc(cc1)N(=O)=O*	*0.84*	*1.39*	*-7.29*
44	*N,N-Diethyl aniline*	*91-66-7*	*CCN(c1ccccc1)CC*	*1.6*	*3.31*	*-2.11*
45	*Indole*	*120-72-9*	*c1ccc2c(c1)[nH]cc2*	*1.4*	*2.14*	*-4.67*
46	*Diazepam*	*439-14-5*	*Clc1ccc2c(c1)C(=NCC(=O)N2C)c1ccccc1*	*2.01*	*2.82*	*-8.59*

[a] Data Sources: [1–5]

Table S 2: Chemicals with their experimental values of $logK_{fish}$ and values of $logK_{ow}$ and $logK_{aw}$ used to calibrate fish muscle protein-water 2p-LFER model. [a]

S. No	Chemicals	CAS-RN	SMILES	$logK_{fish}$	$logK_{ow}$	$logK_{aw}$
1	n-Nonane	111-84-2	CCCCCCCCC	3.68	5.65	2.14

S. No	Chemicals	CAS-RN	SMILES	$logK_{fish}$	$logK_{ow}$	$logK_{aw}$
2	Cyclooctane	292-64-8	C1CCCCCCC1	2.82	4.45	1.18
3	Cycloheptane	291-64-5	C1CCCCCC1	2.33	4.00	0.58
4	Non-1-ene	124-11-8	CCCCCCCC=C	3.52	5.15	1.51
5	Hept-1-yne	628-71-7	CCCCCC#C	1.62	3.44	0.46
6	1-Chlorooctane	111-85-3	CCCCCCCCCl	2.88	3.64	0.19
7	Di-n-butyl ether	142-96-1	CCCCOCCCC	1.52	3.21	-0.61
8	Octan-2-one	111-13-7	CCCCCCC(=O)C	1.35	2.37	-2.11
9	Nonan-2-one	821-55-6	CCCCCCCC(=O)C	1.51	3.14	-1.82
10	Decan-2-one	693-54-9	CCCCCCCCC(=O)C	1.96	3.73	-1.91
11	Tri-n-butyl phosphate	126-73-8	CCCCOP(=O)(OCCCC)OCCCC	1.83	4.00	-4.24
12	Hexan-1-ol	111-27-3	CCCCCCO	0.55	2.03	-3.16
13	Octan-1-ol	111-87-5	CCCCCCCCO	1.55	3.00	-3.00
14	Nonan-1-ol	143-08-8	CCCCCCCCCO	1.99	3.77	-2.90
15	4-Ethyl-3-hexanol	19780-44-0	CCC(C(CC)O)CC	0.75	2.78	-2.85
16	n-Propylbenzene	103-65-1	CCCc1ccccc1	1.95	3.69	-0.37
17	n-Pentylbenzene	538-68-1	CCCCCc1ccccc1	2.77	4.90	0.02
18	n-Hexylbenzene	1077-16-3	CCCCCCc1ccccc1	3.24	5.52	0.07
19	1,2,4-Trimethylbenzene	95-63-6	Cc1ccc(c(c1)C)C	1.87	3.63	-0.60

S. No	Chemicals	CAS-RN	SMILES	$logK_{fish}$	$logK_{ow}$	$logK_{aw}$
20	1,2-Dichlorobenzene	95-50-1	Clc1ccccc1Cl	2.04	3.43	-1.11
21	1,2,3,4-Tetrachlorobenzene	634-66-2	Clc1c(Cl)ccc(c1Cl)Cl	2.86	4.60	-1.51
22	1,2,4,5-Tetrachlorobenzene	95-94-3	Clc1cc(Cl)c(cc1Cl)Cl	3	4.64	-1.39
23	Pentachlorobenzene	608-93-5	Clc1cc(Cl)c(c(c1Cl)Cl)Cl	3.41	5.17	-1.54
24	1,4-Dibromobenzene	106-37-6	Brc1ccc(cc1)Br	2.28	3.79	-1.44
25	Dibenzothiophene	132-65-0	c1ccc2c(c1)sc1c2cccc1	3.46	4.38	-2.86
26	Phenanthrene	85-01-8	c1ccc2c(c1)c1ccccc1cc2	3.25	4.46	-2.76
27	Pyrene	129-00-0	c1cc2ccc3c4c2c(c1)ccc4ccc3	3.76	4.88	-3.31
28	Chrysene	218-01-9	c1ccc2c(c1)c1ccc3c(c1cc2)cccc3	4.41	5.81	-3.67
29	Benzo[a]pyrene	50-32-8	c1ccc2c(c1)c1ccc3c4c1c(c2)ccc4ccc3	4.97	6.13	-4.73
30	Valerophenone	1009-14-9	CCCCC(=O)c1ccccc1	1.75	3.29	-2.98
31	Benzophenone	119-61-9	O=C(c1ccccc1)c1ccccc1	1.99	3.18	-4.46
32	Ethyl benzoate	93-89-0	CCOC(=O)c1ccccc1	1.27	2.64	-2.52
33	di-n-propyl phthalate	131-16-8	CCCOC(=O)c1ccccc1C(=O)OCCC	1.92	3.27	-4.78
34	2-Nitrotoluene	88-72-2	O=N(=O)c1ccccc1C	1.32	2.30	-3.29
35	2,4-Dinitrotoluene	121-14-2	O=N(=O)c1ccc(c(c1)N(=O)=O)C	1.27	1.98	-5.66
36	1-Nitronaphthalene	86-57-7	O=N(=O)c1cccc2c1cccc2	2.16	3.19	-4.14

S. No	Chemicals	CAS-RN	SMILES	$logK_{fish}$	$logK_{ow}$	$logK_{aw}$
37	4-n-Propylphenol	645-56-7	CCCc1ccc(cc1)O	1.63	3.20	-4.33
38	4-Fluorophenol	371-41-5	Oc1ccc(cc1)F	1.1	1.77	-4.54
39	3-Chlorophenol	108-43-0	Oc1cccc(c1)Cl	1.55	2.50	-4.85
40	4-Chlorophenol	106-48-9	Oc1ccc(cc1)Cl	1.45	2.39	-4.59
41	4-Bromophenol	106-41-2	Oc1ccc(cc1)Br	1.62	2.59	-5.21
42	4-Iodophenol	540-38-5	Oc1ccc(cc1)I	1.84	2.91	-5.41
43	N,N-Diethylaniline	91-66-7	CCN(c1ccccc1)CC	1.52	3.31	-2.11
44	Indole	120-72-9	c1ccc2c(c1)[nH]cc2	1.97	2.14	-4.67
45	Diazepam	439-14-5	Clc1ccc2c(c1)C(=NCC(=O)N2C)c1ccccc1	2.1	2.82	-8.59

[a] Data Sources: [1–5]

Table S 3: Chemicals with their experimental values of $logK_{BSA}$ and values of $logK_{ow}$ and $logK_{aw}$ used to calibrate bovine serum albumin-water 2p-LFER model. [a]

S. No	Chemicals	CAS-RN	SMILES	$logK_{BSA}$	$logK_{ow}$	$logK_{aw}$	E	S	A	B	V	L
1	n-hexane	110-54-3	CCCCCC	3.09	3.90	1.88	0	0	0	0	0.954	2.668
2	n-heptane	142-82-5	CCCCCCC	3.59	4.66	1.92	0	0	0	0	1.0949	3.173
3	n-octane	111-65-9	CCCCCCCC	4.01	5.18	2.13	0	0	0	0	1.2358	3.677
4	n-nonane	111-84-2	CCCCCCCCC	4.45	5.65	2.15	0	0	0	0	1.3767	4.182
5	cyclohexane	110-82-7	C1CCCCC1	2.01	3.44	0.80	0.31	0.1	0	0	0.8454	2.964

S. No	Chemicals	CAS-RN	SMILES	$logK_{BSA}$	$logK_{ow}$	$logK_{aw}$	E	S	A	B	V	L
6	cycloheptane	291-64-5	C1CCCCCC1	2.52	4.00	0.59	0.35	0.1	0	0	0.9863	3.704
7	cyclooctane	292-64-8	C1CCCCCCC1	2.98	4.45	1.18	0.41	0.1	0	0	1.1272	4.329
8	1-nonene	124-11-8	CCCCCCCC=C	4.22	5.15	1.52	0.09	0.08	0	0.07	1.3337	4.073
9	1-heptyne	628-71-7	CCCCCC#C	2.49	3.44	0.47	0.16	0.23	0.13	0.1	1.0089	3
10	1-chlorooctane	111-85-3	CCCCCCCCCl	3.85	4.64	0.20	0.19	0.4	0	0.09	1.3582	4.708
11	tetrachloromethane	56-23-5	ClC(Cl)(Cl)Cl	1.77	2.83	0.06	0.46	0.38	0	0	0.7391	2.823
12	trichloroethene	79-01-6	ClC=C(Cl)Cl	1.88	2.42	-0.39	0.52	0.37	0.08	0.03	0.7146	2.997
13	tetrachloroethene	127-18-4	ClC(=C(Cl)Cl)Cl	2.4	3.40	-0.13	0.64	0.44	0	0	0.837	3.584
14	tribromomethane	75-25-2	BrC(Br)Br	1.95	2.40	-1.65	0.97	0.68	0.15	0.06	0.7745	3.784
15	g-hexachlorocyclohexane	58-89-9	Cl[C@@H]1[C@H](Cl)[C@@H](Cl)[C@@H]([C@@H]([C@H]1Cl)Cl)Cl	2.46	4.14	-3.67	1.45	0.91	0	0.68	1.5798	7.467
16	isoflurane	26675-46-7	FC(OC(C(F)(F)F)Cl)F	1.58	2.06	0.08	-0.24	0.5	0.1	0.1	0.8009	1.576
17	enflurane	13838-16-9	FC(OC(C(Cl)F)(F)F)F	1.59	2.10	0.03	-0.24	0.54	0.01	0.1	0.8009	2.009
18	halothane	151-67-7	ClC(C(F)(F)F)Br	1.62	2.30	-0.07	0.1	0.38	0.15	0.03	0.7409	2.177
19	methoxyflurane	76-38-0	COC(C(Cl)Cl)(F)F	1.77	2.21	-0.81	0.11	0.67	0.17	0.05	0.8702	2.864
20	di-*n*-butyl ether	142-96-1	CCCCOCCCC	2.01	3.21	-0.60	0	0.25	0	0.45	1.2945	3.924
21	di-*n*-pentyl ether	693-65-2	CCCCCOCCCCC	3	4.28	-0.44	0	0.25	0	0.45	1.5763	4.875

S. No	Chemicals	CAS-RN	SMILES	$logK_{BSA}$	$logK_{ow}$	$logK_{aw}$	E	S	A	B	V	L
22	2-octanone	111-13-7	CCCCCCC(=O)C	2.09	2.37	-2.11	0.11	0.68	0	0.51	1.2515	4.257
23	2-nonanone	821-55-6	CCCCCCCC(=O)C	2.48	3.14	-1.82	0.12	0.68	0	0.51	1.3924	4.735
24	2-decanone	693-54-9	CCCCCCCCC(=O)C	2.88	3.73	-1.91	0.11	0.68	0	0.51	1.5333	5.245
25	1-nitrooctane	629-37-8	CCCCCCCCN(=O)=O	3.38	3.57	-1.67	0.19	0.95	0	0.29	1.41	5.154
26	tri-*n*-butyl phosphate	126-73-8	CCCCOP(=O)(OCCCC)OCCCC	2.47	4.00	-4.23	-0.1	0.71	0	1.26	2.2388	7.539
27	benzene	71-43-2	c1ccccc1	1.58	2.13	-0.64	0.61	0.52	0	0.14	0.7164	2.786
28	toluene	108-88-3	Cc1ccccc1	2.26	2.73	-0.56	0.6	0.52	0	0.14	0.8573	3.325
29	ethylbenzene	100-41-4	CCc1ccccc1	2.7	3.15	-0.48	0.61	0.51	0	0.15	0.9982	3.778
30	n-propylbenzene	103-65-1	CCCc1ccccc1	2.95	3.69	-0.36	0.6	0.5	0	0.15	1.1391	4.23
31	styrene	100-42-5	C=Cc1ccccc1	2.76	2.95	-0.94	0.85	0.65	0	0.16	0.9552	3.856
32	chlorobenzene	108-90-7	Clc1ccccc1	2.32	2.84	-0.89	0.72	0.65	0	0.07	0.8388	3.657
33	1,2-dichlorobenzene	95-50-1	Clc1ccccc1Cl	3.03	3.43	-1.10	0.87	0.78	0	0.04	0.9612	4.518
34	1,2,4-trimethylbenzene	95-63-6	Cc1ccc(c(c1)C)C	3.35	3.63	-0.59	0.68	0.56	0	0.19	1.1391	4.441
35	1,4-dibromobenzene	106-37-6	Brc1ccc(cc1)Br	3.97	3.79	-1.43	1.15	0.86	0	0.04	1.0664	5.324
36	1,2,4-trichlorobenzene	120-82-1	Clc1ccc(c(c1)Cl)Cl	3.6	4.02	-1.23	0.98	0.81	0	0	1.0836	5.248

S. No	Chemicals	CAS-RN	SMILES	$logK_{BSA}$	$logK_{ow}$	$logK_{aw}$	E	S	A	B	V	L
37	1,2,3,4-tetrachlorobenzene	634-66-2	Clc1c(Cl)ccc(c1Cl)Cl	4.21	4.60	-1.50	1.18	0.92	0	0	1.206	6.171
38	hexafluorobenzene	392-56-3	Fc1c(F)c(F)c(c(c1F)F)F	1.55	2.55	0.35	0.09	0.56	0	0.01	0.8226	2.345
39	methylpentafluorobenzene	771-56-2	Fc1c(C)c(F)c(c(c1F)F)F	2.32	3.07	0.17	0.06	0.59	0	0.01	0.9458	3.244
40	indene	95-13-6	c1ccc2c(c1)C=CC2	2.92	2.92	-1.65	1	0.77	0	0.2	0.9875	4.559
41	naphthalene	91-20-3	c1ccc2c(c1)cccc2	3.56	3.30	-1.74	1.34	0.92	0	0.2	1.0854	5.161
42	dibenzofuran	132-64-9	c1ccc2c(c1)oc1c2cccc1	3.79	4.12	-2.05	1.41	1.02	0	0.17	1.2743	6.716
43	dibenzothiophene	132-65-0	c1ccc2c(c1)sc1c2cccc1	4.16	4.38	-2.85	1.96	1.31	0	0.18	1.3791	7.588
44	phenanthrene	85-01-8	c1ccc2c(c1)c1ccccc1cc2	4.15	4.46	-2.75	2.06	1.29	0	0.26	1.4544	7.632
45	fluoranthene	206-44-0	c1ccc2c(c1)c1cccc3c1c2ccc3	4.28	5.16	-3.43	2.38	1.55	0	0.24	1.5846	8.827
46	pyrene	129-00-0	c1cc2ccc3c4c2c(c1)ccc4ccc3	4.76	4.88	-3.30	2.81	1.71	0	0.28	1.5846	8.833
47	chrysene	218-01-9	c1ccc2c(c1)c1ccc3c(c1cc2)cccc3	4.46	5.81	-3.66	3.03	1.73	0	0.33	1.8234	10.334
48	benzo[*b*]fluoranthene	205-99-2	c1ccc2c(c1)c1cc3ccccc3c3c1c2ccc3	4.42	5.78	-4.56	3.19	1.82	0	0.4	1.9536	11.632
49	benzo[*ghi*]perylene	191-24-2	c1cc2ccc3c4c2c(c1)c1cccc2c1c4c(cc3)cc2	4.76	6.76	-6.59	4.07	1.9	0	0.45	2.0838	13.264
50	anisole	100-66-3	COc1ccccc1	2.16	2.11	-1.70	0.71	0.75	0	0.29	0.916	3.89
51	valerophenone	1009-14-9	CCCCC(=O)c1ccccc1	2.7	3.29	-2.98	0.8	0.95	0	0.5	1.4366	5.9
52	benzophenone	119-61-9	O=C(c1ccccc1)c1ccccc1	2.62	3.18	-4.46	1.45	1.5	0	0.5	1.4808	6.852

S. No	Chemicals	CAS-RN	SMILES	$logK_{BSA}$	$logK_{ow}$	$logK_{aw}$	E	S	A	B	V	L
53	di-*n*-propyl phthalate	131-16-8	CCCOC(=O)c1ccccc1C(=O)OCCC	2.84	3.27	-4.77	0.71	1.28	0	0.94	1.9924	8.271
54	2-nitrotoluene	88-72-2	O=N(=O)c1ccccc1C	2.12	2.30	-3.28	0.87	1.11	0	0.28	1.0315	4.878
55	2,4-dinitrotoluene	121-14-2	O=N(=O)c1ccc(c(c1)N(=O)=O)C	1.73	1.98	-5.65	1.15	1.84	0	0.56	1.2057	6.272
56	1-nitronaphthalene	86-57-7	O=N(=O)c1cccc2c1cccc2	3.17	3.19	-4.13	1.6	1.59	0	0.29	1.2596	7.056
57	4-nitroanisole	100-17-4	COc1ccc(cc1)N(=O)=O	2.48	2.03	-4.67	0.98	1.47	0	0.46	1.0902	5.735
58	N,*N*-diethylaniline	91-66-7	CCN(c1ccccc1)CC	2.27	3.31	-2.10	0.95	0.8	0	0.41	1.3798	5.287
59	1-hexanol	111-27-3	CCCCCCO	1.64	2.03	-3.15	0.21	0.42	0.37	0.48	1.0127	3.61
60	1-heptanol	111-70-6	CCCCCCCO	2.18	2.62	-3.11	0.21	0.42	0.37	0.48	1.1536	4.115
61	1-octanol	111-87-5	CCCCCCCCO	2.74	3.00	-2.99	0.2	0.42	0.37	0.48	1.2945	4.619
62	1-nonanol	143-08-8	CCCCCCCCCO	3.1	3.77	-2.89	0.19	0.42	0.37	0.48	1.4354	5.124
63	4-ethyl-3-hexanol	19780-44-0	CCC(C(CC)O)CC	1.48	2.78	-2.85	0.17	0.36	0.33	0.57	1.2945	4.177
64	4-chlorobenzyl alcohol	873-76-7	OCc1ccc(cc1)Cl	2.1	1.96	-5.02	0.91	0.96	0.4	0.5	1.0384	4.938
65	4-*n*-propylphenol	645-56-7	CCCc1ccc(cc1)O	2.59	3.20	-4.32	0.79	0.88	0.55	0.37	1.1978	5.185
66	2-phenylphenol	90-43-7	Oc1ccccc1c1ccccc1	2.62	3.09	-4.36	1.55	1.4	0.56	0.49	1.3829	7.227
67	4-fluorophenol	371-41-5	Oc1ccc(cc1)F	1.57	1.77	-4.53	0.67	0.97	0.63	0.23	0.7928	3.844
68	3-chlorophenol	108-43-0	Oc1cccc(c1)Cl	2.35	2.50	-4.84	0.91	1.06	0.69	0.15	0.8975	4.773
69	4-chlorophenol	106-48-9	Oc1ccc(cc1)Cl	2.43	2.39	-4.58	0.92	1.08	0.67	0.21	0.8975	4.775

S. No	Chemicals	CAS-RN	SMILES	$logK_{BSA}$	$logK_{ow}$	$logK_{aw}$	E	S	A	B	V	L
70	4-bromophenol	106-41-2	Oc1ccc(cc1)Br	2.81	2.59	-5.20	1.08	1.17	0.67	0.2	0.9501	5.135
71	4-iodophenol	540-38-5	Oc1ccc(cc1)I	3.41	2.91	-5.41	1.38	1.22	0.68	0.2	1.0333	5.492
72	bisphenol A	80-05-7	CC(c1ccc(cc1)O)(c1ccc(cc1)O)C	2.88	3.32	-10.56	1.61	1.56	0.99	0.91	1.8643	9.603
73	4-nitroaniline	100-01-6	Nc1ccc(cc1)N(=O)=O	1.69	1.39	-7.28	1.22	1.91	0.42	0.38	0.9904	6.343
74	2-chloroaniline	95-51-2	Nc1ccccc1Cl	1.95	1.90	-3.65	1.03	0.92	0.25	0.31	0.9386	4.674
75	4-iodoaniline	540-37-4	Nc1ccc(cc1)I	2.95	2.34	-5.14	1.53	1.28	0.31	0.4	1.0744	5.695
76	4-aminobiphenyl	92-67-1	Nc1ccc(cc1)c1ccccc1	2.55	2.86	-5.83	1.57	1.48	0.26	0.48	1.424	7.698
77	indole	120-72-9	c1ccc2c(c1)[nH]cc2	2.25	2.14	-4.66	1.2	1.12	0.44	0.22	0.9464	5.31
78	carbazole	86-74-8	c1ccc2c(c1)[nH]c1c2cccc1	3.52	3.72	-5.32	1.79	2.12	0.09	0.1	1.3154	8.002
79	metolachlor	51218-45-2	COCC(N(c1c(C)cccc1CC)C(=O)CCl)C	1.74	2.90	-6.43	1.11	1.46	0	1.27	2.2811	8.75
80	atrazine	1912-24-9	CCNc1nc(NC(C)C)nc(n1)Cl	1.77	2.61	-7.01	1.22	1.29	0.17	1.01	1.6196	7.783
81	diazepam	439-14-5	Clc1ccc2c(c1)C(=NCC(=O)N2C)c1ccccc1	2.68	2.82	-8.59	2.08	1.55	0	1.28	2.0739	10.48
82	estrone	53-16-7	Oc1ccc2c(c1)CC[C@@H]1[C@@H]2CC[C@]2([C@H]1CCC2=O)C	2.69	3.13	-10.43	1.73	2.05	0.5	1.08	2.1558	10.78
83	endosulfan α	115-29-7	O=S1OCC2C(CO1)C1(C(C2(Cl)C(=C1Cl)Cl)(Cl)Cl)Cl	3.24	3.83	-2.57	2.44	0.95	0.35	1.2	2.0819	9.035

[a] Data Sources: [1–5]

Table S 4: Chemicals with their experimental values of $\boldsymbol{logK_{pw}}$ and values of $\boldsymbol{logK_{ow}}$ and $\boldsymbol{logK_{aw}}$ used to calibrate combined chicken and fish muscle protein-water 2p-LFER model. [a]

S. No	Chemicals	CAS-RN	SMILES	$logK_{pw}$	$logK_{ow}$	$logK_{aw}$	E	S	A	B	V	L
1	*n-Nonane*	*111-84-2*	*CCCCCCCCC*	*3.77*	*5.65*	*2.14*	*0*	*0*	*0*	*0*	*1.3767*	*4.182*
2	*Cyclooctane*	*292-64-8*	*C1CCCCCCC1*	*2.88*	*4.45*	*1.18*	*0.41*	*0.1*	*0*	*0*	*1.1272*	*4.329*
3	*Cycloheptane*	*291-64-5*	*C1CCCCCC1*	*2.25*	*4.00*	*0.58*	*0.35*	*0.1*	*0*	*0*	*0.9863*	*3.704*
4	*Non-1-ene*	*124-11-8*	*CCCCCCCC=C*	*3.53*	*5.15*	*1.51*	*0.09*	*0.08*	*0*	*0.07*	*1.3337*	*4.073*
5	*Hept-1-yne*	*628-71-7*	*CCCCCC#C*	*1.69*	*3.44*	*0.46*	*0.16*	*0.23*	*0.13*	*0.1*	*1.0089*	*3*
6	*1-Chlorooctane*	*111-85-3*	*CCCCCCCCCl*	*2.91*	*3.64*	*0.19*	*0.19*	*0.4*	*0*	*0.09*	*1.3582*	*4.708*
7	*Di-n-butyl ether*	*142-96-1*	*CCCCOCCCC*	*1.62*	*3.21*	*-0.61*	*0*	*0.25*	*0*	*0.45*	*1.2945*	*3.924*
8	*Octan-2-one*	*111-13-7*	*CCCCCCC(=O)C*	*1.46*	*2.37*	*-2.11*	*0.11*	*0.68*	*0*	*0.51*	*1.2515*	*4.257*
9	*Nonan-2-one*	*821-55-6*	*CCCCCCCC(=O)C*	*1.67*	*3.14*	*-1.82*	*0.12*	*0.68*	*0*	*0.51*	*1.3924*	*4.735*
10	*Decan-2-one*	*693-54-9*	*CCCCCCCCC(=O)C*	*2.04*	*3.73*	*-1.91*	*0.11*	*0.68*	*0*	*0.51*	*1.5333*	*5.245*
11	*1-Nitrohexane*	*646-14-0*	*CCCCCCN(=O)=O*	*1.34*	*2.70*	*-2.03*	*0.2*	*0.95*	*0*	*0.29*	*1.1282*	*4.416*
12	*Tri-n-butyl phosphate*	*126-73-8*	*CCCCOP(=O)(OCCCC)OCCCC*	*1.76*	*4.00*	*-4.24*	*-0.1*	*0.71*	*0*	*1.26*	*2.2388*	*7.539*
13	*Hexan-1-ol*	*111-27-3*	*CCCCCCO*	*0.55*	*2.03*	*-3.16*	*0.21*	*0.42*	*0.37*	*0.48*	*1.0127*	*3.61*
14	*Octan-1-ol*	*111-87-5*	*CCCCCCCCO*	*1.49*	*3.00*	*-3.00*	*0.2*	*0.42*	*0.37*	*0.48*	*1.2945*	*4.619*

S. No	Chemicals	CAS-RN	SMILES	$logK_{pw}$	$logK_{ow}$	$logK_{aw}$	E	S	A	B	V	L
15	Nonan-1-ol	143-08-8	CCCCCCCCCO	1.98	3.77	-2.90	0.19	0.42	0.37	0.48	1.4354	5.124
16	4-Ethyl-3-hexanol	19780-44-0	CCC(C(CC)O)CC	0.75	2.78	-2.85	0.17	0.36	0.33	0.57	1.2945	4.177
17	n-Propylbenzene	103-65-1	CCCc1ccccc1	1.97	3.69	-0.37	0.6	0.5	0	0.15	1.1391	4.23
18	n-Pentylbenzene	538-68-1	CCCCCc1ccccc1	2.74	4.90	0.02	0.59	0.51	0	0.15	1.4209	5.23
19	n-Hexylbenzene	1077-16-3	CCCCCCc1ccccc1	3.22	5.52	0.07	0.59	0.5	0	0.15	1.5618	5.72
20	1,2,4-Trimethylbenzene	95-63-6	Cc1ccc(c(c1)C)C	1.89	3.63	-0.60	0.68	0.56	0	0.19	1.1391	4.441
21	1,2-Dichlorobenzene	95-50-1	Clc1ccccc1Cl	2.03	3.43	-1.11	0.87	0.78	0	0.04	0.9612	4.518
22	1,2,3,4-Tetrachlorobenzene	634-66-2	Clc1c(Cl)ccc(c1Cl)Cl	2.83	4.60	-1.51	1.18	0.92	0	0	1.206	6.171
23	1,2,4,5-Tetrachlorobenzene	95-94-3	Clc1cc(Cl)c(cc1Cl)Cl	2.97	4.64	-1.39	1.16	0.86	0	0	1.206	5.926
24	Pentachlorobenzene	608-93-5	Clc1cc(Cl)c(c(c1Cl)Cl)Cl	3.38	5.17	-1.54	1.33	0.92	0.06	0	1.3284	6.63
25	1,4-Dibromobenzene	106-37-6	Brc1ccc(cc1)Br	2.23	3.79	-1.44	1.15	0.86	0	0.04	1.0664	5.324
26	Dibenzothiophene	132-65-0	c1ccc2c(c1)sc1c2cccc1	3.43	4.38	-2.86	1.96	1.31	0	0.18	1.3791	7.588
27	Phenanthrene	85-01-8	c1ccc2c(c1)c1ccccc1cc2	3.20	4.46	-2.76	2.06	1.29	0	0.26	1.4544	7.632
28	Pyrene	129-00-0	c1cc2ccc3c4c2c(c1)ccc4ccc3	3.72	4.88	-3.31	2.81	1.71	0	0.28	1.5846	8.833
29	Chrysene	218-01-9	c1ccc2c(c1)c1ccc3c(c1cc2)cccc3	4.47	5.81	-3.67	3.03	1.73	0	0.33	1.8234	10.334

S. No	Chemicals	CAS-RN	SMILES	$logK_{pw}$	$logK_{ow}$	$logK_{aw}$	E	S	A	B	V	L
30	Benzo[a]pyrene	50-32-8	c1ccc2c(c1)c1ccc3c4c1c(c2)ccc4ccc3	4.93	6.13	-4.73	3.63	1.98	0	0.44	1.9536	11.736
31	4-Nitroanisole	100-17-4	COc1ccc(cc1)N(=O)=O	1.38	2.03	-4.21	0.98	1.49	0	0.37	1.0902	5.62
32	Valerophenone	1009-14-9	CCCCC(=O)c1ccccc1	1.75	3.29	-2.98	0.8	0.95	0	0.5	1.4366	5.9
33	Benzophenone	119-61-9	O=C(c1ccccc1)c1ccccc1	2.00	3.18	-4.46	1.45	1.5	0	0.5	1.4808	6.852
34	Ethyl benzoate	93-89-0	CCOC(=O)c1ccccc1	1.27	2.64	-2.52	0.69	0.85	0	0.46	1.2135	5.075
35	di-n-propyl phthalate	131-16-8	CCCOC(=O)c1ccccc1C(=O)OCCC	1.92	3.27	-4.78	0.71	1.28	0	0.94	1.9924	8.271
36	2-Nitrotoluene	88-72-2	O=N(=O)c1ccccc1C	1.30	2.30	-3.29	0.87	1.11	0	0.28	1.0315	4.878
37	2,4-Dinitrotoluene	121-14-2	O=N(=O)c1ccc(c(c1)N(=O)=O)C	1.25	1.98	-5.66	1.15	1.58	0	0.49	1.2057	6.258
38	1-Nitronaphthalene	86-57-7	O=N(=O)c1cccc2c1cccc2	2.16	3.19	-4.14	1.6	1.59	0	0.29	1.2596	7.056
39	4-n-Propylphenol	645-56-7	CCCc1ccc(cc1)O	1.66	3.20	-4.33	0.79	0.88	0.55	0.37	1.1978	5.185
40	4-Fluorophenol	371-41-5	Oc1ccc(cc1)F	1.14	1.77	-4.54	0.67	0.97	0.63	0.23	0.7928	3.844
41	3-Chlorophenol	108-43-0	Oc1cccc(c1)Cl	1.56	2.50	-4.85	0.91	1.06	0.69	0.15	0.8975	4.773
42	4-Chlorophenol	106-48-9	Oc1ccc(cc1)Cl	1.50	2.39	-4.59	0.92	1.08	0.67	0.21	0.8975	4.775
43	4-Bromophenol	106-41-2	Oc1ccc(cc1)Br	1.66	2.59	-5.21	1.08	1.17	0.67	0.2	0.9501	5.135
44	4-Iodophenol	540-38-5	Oc1ccc(cc1)I	1.89	2.91	-5.41	1.38	1.22	0.68	0.2	1.0333	5.492
45	2-Phenylphenol	90-43-7	Oc1ccccc1c1ccccc1	2.00	3.09	-4.37	1.55	1.4	0.56	0.49	1.3829	7.22
46	4-Chloroaniline	106-47-8	Nc1ccc(cc1)Cl	1.19	1.83	-4.32	1.06	1.13	0.3	0.31	0.9386	4.889
47	4-Iodoaniline	540-37-4	Nc1ccc(cc1)I	1.66	2.34	-5.14	1.53	1.28	0.31	0.4	1.0744	5.695

S. No	Chemicals	CAS-RN	SMILES	$logK_{pw}$	$logK_{ow}$	$logK_{aw}$	E	S	A	B	V	L
48	*4-Nitroaniline*	*100-01-6*	*Nc1ccc(cc1)N(=O)=O*	*0.84*	*1.39*	*-7.29*	*1.22*	*1.91*	*0.42*	*0.38*	*0.9904*	*6.343*
49	*N,N-Diethylaniline*	*91-66-7*	*CCN(c1ccccc1)CC*	*1.56*	*3.31*	*-2.11*	*0.95*	*0.8*	*0*	*0.41*	*1.3798*	*5.287*
50	*Indole*	*120-72-9*	*c1ccc2c(c1)[nH]cc2*	*1.69*	*2.14*	*-4.67*	*1.2*	*1.12*	*0.44*	*0.22*	*0.9464*	*5.31*
51	*Diazepam*	*439-14-5*	*Clc1ccc2c(c1)C(=NCC(=O)N2C)c1ccccc1*	*2.06*	*2.82*	*-8.59*	*2.08*	*1.55*	*0*	*1.28*	*2.0739*	*10.48*

[a] Data Sources[1–6]

Table S 5: Diversity of data for $logK_{pw}$.

Variable	Observations	Obs. with missing data	Minimum	Maximum	Mean	Std. deviation
$logK_{pw}$	51	0	0.550	4.925	2.119	0.939
$\log K_{ow}$	51	0	1.390	6.130	3.456	1.147
logKaw	51	0	-8.590	2.143	-2.769	2.252

Table S 6: Diversity of data for $logK_{BSA}$.

Variable	Observations	Obs. with missing data	Minimum	Maximum	Mean	Std. deviation
$logK_{BSA}$	83	0	1.480	4.760	2.764	0.867
$\log K_{ow}$	83	0	1.390	6.763	3.314	1.059
logKaw	83	0	-10.560	2.151	-2.563	2.703

Table S 7: Comparison of Experimental and 2p-LFER Predicted Partition Coefficients for ionizable PFAS Compounds.

**Chemical**	**log K_{ow}**	**log K_{aw}**	**[a]log K_{BSA} expt**	**log K_{BSA} pred**	**[b]log K_{pw} expt**	**log K_{pw} pred**
**PFBA**	1.56	-2.93	2.52	1.38	NA	0.52
**PFHxA**	3.99	-2.12	3.43	3.26	0.64	2.51
**PFHpA**	4.4	-1.79	4.02	3.56	1.30	2.83
**PFNA**	4.61	-0.97	4.32	3.68	2.17	2.93
**PFOA**	4.67	-1.41	4.2	3.75	1.61	3.02
**HFPO-DA**	4.83	-1.43	3.06	3.88	0.80	3.16
**PFDA**	5.44	-0.53	4.73	4.31	2.96	3.60
**PFHxS**	5.49	-3.94	4.81	4.53	1.73	3.95
**PFUnDA**	5.65	-0.15	4.6	4.46	3.42	3.74
**PFBS**	4.09	-4.89	3.2	3.48	0.74	2.85
**PFECHS**	5.78	-4.1	4.55	4.77	2.57	4.22
**PFOS**	6.84	-3.04	4.67	5.55	2.94	5.02
**9Cl-PF3ONS**	7.42	-2.45	5.01	5.98	3.49	5.46
**RMSE**				**0.61**		**1.64**

[a] The values of experimental logK_{BSA} were extracted from Allendorf et al., 2019.[7]

[b] The values of experimental $logK_{PW}$ were extracted from Allendorf et al., 2021.[8]

Table S 8: List of neutral fluorotelomer PFAS Compounds.[a]

S. No	PFAS	*S*	*A*	*B*	*V*	*L*	$\log K_{ow}$	logKaw	logKmuscle ASM	logKmuscle 2pLFER	logKalbumin ASM	logKalbumin 2pLFER	logKlipid	logKphospholipid
1	3:1FTOH	0.30	0.84	0.14	0.995	1.542	2.34	-2.00	1.23	1.10	2.33	1.95	0.74	2.05
2	3:3FTOH	0.53	0.52	0.39	1.277	2.710	2.40	-2.36	1.22	1.18	2.21	2.02	1.18	2.02
3	4:2FTOH	0.35	0.60	0.31	1.352	2.421	2.97	-1.56	1.66	1.59	2.62	2.42	1.63	2.46
4	4:4FTOH	0.52	0.51	0.44	1.634	3.499	3.44	-2.01	2.08	2.03	2.93	2.82	2.18	2.85
5	6:2FTOH	0.35	0.60	0.31	1.785	2.997	4.26	-0.73	2.77	2.61	3.58	3.40	2.83	3.49
6	7:1FTOH	0.30	0.84	0.14	1.860	2.715	4.93	-0.36	3.45	3.15	4.25	3.90	3.14	4.12
7	8:2FTOH	0.35	0.60	0.31	2.217	3.554	5.54	0.10	3.87	3.63	4.53	4.36	4.01	4.51
8	10:2FTOH	0.35	0.60	0.31	2.650	4.117	6.83	0.94	4.97	4.65	5.48	5.33	5.20	5.54
9	12:2FTOH	0.35	0.60	0.31	3.082	4.682	8.11	1.77	6.08	5.66	6.44	6.30	6.39	6.56
10	5:2sFTOH	0.39	0.62	0.19	1.568	2.296	3.79	-0.52	2.44	2.19	3.35	3.01	2.39	3.18
11	EtFHxSE	1.00	0.55	0.68	2.447	5.685	4.83	-3.30	3.49	3.33	4.06	3.98	3.21	3.97
12	EtFOSE	1.00	0.55	0.68	2.880	6.253	6.11	-2.47	4.60	4.35	5.02	4.95	4.40	5.00
13	MeFBSE	1.00	0.55	0.68	1.874	4.769	3.05	-4.32	1.98	1.91	2.75	2.63	1.54	2.53
14	MeFOSE	1.00	0.55	0.68	2.739	5.909	5.63	-2.66	4.19	3.96	4.66	4.58	3.92	4.58
15	PFBSA	0.96	1.15	0.23	1.392	3.607	2.89	-4.92	2.15	1.83	3.11	2.54	0.68	2.74
16	PFHxSA	0.96	1.15	0.23	1.825	4.189	4.19	-4.09	3.26	2.86	4.07	3.52	1.88	3.78
17	PFOSA	0.96	1.15	0.23	2.257	4.744	5.47	-3.26	4.36	3.87	5.02	4.48	3.06	4.80
18	MeFBSA	0.88	0.74	0.24	1.533	3.566	3.37	-2.92	2.36	2.06	3.28	2.81	1.82	3.08
19	MeFHxSA	0.88	0.74	0.24	1.966	4.135	4.65	-2.09	3.47	3.07	4.24	3.77	3.01	4.11
20	MeFOSA	0.88	0.74	0.24	2.398	4.703	5.94	-1.26	4.57	4.09	5.19	4.75	4.20	5.13
21	EtFHxSA	0.88	0.74	0.24	2.107	4.400	5.11	-1.86	3.85	3.44	4.57	4.13	3.45	4.48
22	EtFOSA	0.88	0.74	0.24	2.539	4.967	6.39	-1.03	4.96	4.45	5.52	5.09	4.63	5.50
23	PFBI	0.07	0	0	1.270	1.939	4.14	2.75	2.35	2.19	3.33	3.12	4.09	3.61
24	PFHxI	0.07	0	0	1.702	2.508	5.42	3.58	3.45	3.20	4.28	4.08	5.28	4.64
25	PFHpI	0.07	0	0	1.919	2.792	6.07	4.00	4.01	3.72	4.76	4.57	5.87	5.15

26	PFOI	0.07	0	0	2.135	3.068	6.71	4.42	4.56	4.22	5.24	5.05	6.46	5.66
27	PFDI	0.07	0	0	2.568	3.623	7.99	5.25	5.66	5.24	6.19	6.02	7.65	6.68
28	4:2FTI	0.32	0.00	0.17	1.552	3.325	4.47	1.46	2.72	2.59	3.58	3.44	4.34	3.91
29	6:1FTI	0.27	0.13	0.11	1.843	3.382	5.45	2.09	3.60	3.37	4.37	4.18	5.03	4.69
30	6:1FTI-7H	0.63	0.24	0.14	1.806	3.887	4.95	0.46	3.41	3.09	4.20	3.88	4.33	4.40
31	6:2FTI	0.32	0	0.17	1.984	3.892	5.76	2.29	3.82	3.61	4.53	4.42	5.53	4.93
32	8:2FTI	0.32	0	0.17	2.417	4.455	7.04	3.12	4.93	4.62	5.49	5.38	6.72	5.96
33	10:2FTI	0.32	0	0.17	2.849	5.020	8.33	3.96	6.03	5.64	6.44	6.35	7.91	6.98
34	PFN	-0.19	0	0	2.131	1.571	6.42	5.66	4.21	3.86	4.93	4.76	6.01	5.16
35	PFDoD	-0.19	0	0	2.780	2.362	8.32	6.94	5.85	5.36	6.34	6.19	7.76	6.67
36	1,8-DHPFO	0.38	0.24	0	1.839	2.130	5.11	2.59	3.49	3.03	4.33	3.89	4.37	4.36
37	4:2 FTO	0.11	0	0	1.250	1.451	3.82	2.85	2.12	1.91	3.14	2.86	3.70	3.30
38	6:2 FTO	0.11	0	0	1.683	2.022	5.11	3.68	3.23	2.93	4.09	3.83	4.89	4.33
39	8:2 FTO	0.11	0	0	2.116	2.504	6.36	4.56	4.31	3.91	5.02	4.77	6.03	5.32
40	10:2 FTO	0.11	0	0	2.548	3.031	7.63	5.40	5.40	4.92	5.97	5.73	7.20	6.32
41	6:2 FTAC	0.54	0	0.40	2.180	4.098	5.21	1.12	3.45	3.25	4.12	4.05	4.73	4.31
42	8:2 FTAC	0.54	0	0.40	2.613	4.661	6.50	1.95	4.55	4.27	5.07	5.02	5.92	5.33
43	10:2 FTAC	0.54	0	0.40	3.045	5.221	7.78	2.78	5.65	5.29	6.02	5.98	7.10	6.35
44	4:2 FTMAC	0.53	0	0.39	1.888	3.990	4.51	0.49	2.83	2.71	3.59	3.53	4.14	3.80
45	6:2 FTMAC	0.53	0	0.39	2.321	4.553	5.80	1.33	3.93	3.73	4.54	4.50	5.33	4.82
46	8:2 FTMAC	0.53	0	0.39	2.754	5.115	7.08	2.16	5.04	4.75	5.50	5.46	6.52	5.85
47	10:2 FTMAC	0.53	0	0.39	3.186	5.671	8.36	3.00	6.14	5.76	6.45	6.43	7.70	6.87
	RMSE									0.314		0.270		

[a] **Data Source:** [9–11]

Table S 9: List of experimental in vivo and in vitro partitioning data for different tissues and species.[a]

S. No	Tissue	CAS	Chemical	species	blood/plasma/ serum	measured
1	**adipose**	80-07-9	p,p'-dichlorodiphenyl sulfone	rat	blood	2.03
2		2051-62-9	PCB 3	rat	blood	1.48
3		2050-68-2	PCB 15	rat	blood	1.85
4		37680-73 2	PCB 101	rat	blood	1.85
5		35065-27-1	PCB 153	rat	blood	2.6
6		33284-52-5	PCB 80	rat	blood	2.34
7		35694-04-3	PCB 133	rat	blood	2.01
8		38411-22-2	PCB 136	rat	blood	1.95
9		35065-27-1	PCB 153	rat	blood	2.06
10		33979-03 2	PCB 155	rat	blood	1.95
11		439-14-5	diazepam	rat	plasma	1.13
12		938-73-8	ethenzamide	rat	plasma	-0.17
13		2050-68-2	PCB 15	human	plasma	1.95
14		2050-68-2	PCB 15	human	plasma	1.95
15		7012-37-5	PCB 28	human	plasma	2.3
16		35693-99-3	PCB 52	human	plasma	1.9
17		70362-47-9	PCB 48	human	plasma	1.95
18		2437-79-8	PCB 47	human	plasma	2.49

S. No	Tissue	CAS	Chemical	species	blood/plasma/ serum	measured
19		41464-39 5	PCB 44	human	plasma	1.9
20		32690-93 0	PCB 74	human	plasma	2.3
21		32598-10-0	PCB 66	human	plasma	2.2
22		32598-11-1	PCB 70	human	plasma	1.85
23		38379-99-6	PCB 95	human	plasma	1.78
24		37680-73-2	PCB 101	human	plasma	1.7
25		38380-01-7	PCB 99	human	plasma	2.38
26		32598-14-4	PCB 105	human	plasma	2.11
27		31508-00 6	PCB 118	human	plasma	2.25
28		51908-16 8	PCB 146	human	plasma	2.48
29		35065-27 1	PCB 153	human	plasma	2.43
30		39635-35 3	PCB 159	human	plasma	2.56
31		38380-08-4	PCB 156	human	plasma	2.49
32		52663-72-6	PCB 167	human	plasma	2.08
33		35065-28 2	PCB 138	human	plasma	2.04
34		35065-30-6	PCB 170	human	plasma	2.56
35		35065-29-3	PCB 180	human	plasma	2.43
36		52663-68-0	PCB 187	human	plasma	2.57
37		52663-69-1	PCB 183	human	plasma	2.2
38		52663-75 9	PCB 199	human	plasma	2.42
39		72-55 9	P,P'-DDE	human	plasma	2.23
40	**brain**	107-19-7	propargyl alcohol	rat	blood	-0.23
41		107-13-1	acrylonitrile	rat	blood	-0.4
42			1 -(3-fluoropropyl)-2nitroimidazole	rat	blood	-0.24
43			1 -(8-fluorooctyl)-2-nitroimidazole	rat	blood	-0.17

S. No	Tissue	CAS	Chemical	species	blood/plasma/ serum	measured
44		92817-10-2	16alpha-fluoroestradiol	rat	blood	-0.3
45		5400-60-2	4-fluoroantipyrme	rat	blood	-0.05
46		129-81-7	4-iodoantipyrine	rat	blood	-0.1
47		25526-93-6	alovudine	rat	blood	-0.59
48		66893-81 0	alpha-(4-pyridyl- 1 -oxide)-N-tertbutylnitrone	rat	blood	-0.38
49		60-80-0	antipyrine	rat	blood	-0.1
50		84379-13-5	bretazenil	rat	blood	-0.09
51		57924	caffeine	rat	blood	-0.06
52		298-46-4	carbamazepine	rat	blood	0
53		154-93-8	carmustine	rat	blood	-0.52
54		486-56-6	cotinine	rat	blood	-0.32
55		78755-81-4	flumazenil	rat	blood	-0.29
56			fluoromisonidazole	rat	blood	-0.01
57		604-75-1	oxazepam	rat	blood	0.61
58		103-90-2	paracetamol	rat	blood	-0.34
59		611-59-6	paraxanthine	rat	blood	0.06
60		60-80-0	antipyrine	rat	blood	-0.1
61		3376-24-7	phenyl-N-tert-butylnitrone	rat	blood	0.05
62		2078-54-8	propofol	rat	blood	0.48
63		61413-54-5	rolipram	rat	blood	0.61
64		119054-09-0	tertbutylchlorambucil	rat	blood	1
65		5630-53-5	tibolone	rat	blood	0.4
66		7732-18-5	water	rat	blood	-0.04
67		1088-11-5	nordazepam	rat	blood	0.5
68		90058-29-0	alaptide	rat	plasma	-0.26
69		60-80-0	antipyrine	rat	plasma	-0.22

S. No	Tissue	CAS	Chemical	species	blood/plasma/ serum	measured
70		94-25-7	butyl 4-aminobenzoate	rat	plasma	0.42
71		58-08-2	caffeine	rat	plasma	0.12
72		486-56-6	cotinine	rat	plasma	-0.38
73		439-14-5	diazepam	rat	plasma	0.03
74		938-73-8	ethenzamide	rat	plasma	-0.05
75		94-09-7	ethyl 4-aminobenzoate	rat	plasma	0.27
76		846-49-1	lorazepam	rat	plasma	0.47
77		69-65-8	mannitol	rat	plasma	-1.6
78		2078-54-8	propofol	rat	plasma	0.91
79		71195	propyl 4-aminobenzoate	rat	plasma	0.55
80		129-00-0	pyrene	rat	plasma	0.23
81		3056-17-5	stavudine	rat	plasma	-0.48
82		83-67-0	theobromine	rat	plasma	-0.3
83		30516-87-1	zidovudine	rat	plasma	-0.72
84		58-15-1	aminopyrine	rat	serum	0
85		65873	bisphenol A	rat	serum	-0.12
86		439-14-5	diazepam	rat	serum	0.52
87		1622-62-4	ftunitrazepam	rat	serum	0.06
88		846-49-1	lorazepam	rat	serum	0.41
89		604-75-1	oxazepam	rat	serum	0.55
90		54-95-5	pentylenetetrazole	rat	serum	-0.03
91		28911-01-5	triazolam	rat	serum	0.57
92	**liver**		1-(3-fluoropropyl)-2-niooimidazole	rat	blood	0.92
93			1-(8-ftuorooctyl)-2-nitroimidazole	rat	blood	0.47
94		2051-62-9	PCB3	rat	blood	0

S. No	Tissue	CAS	Chemical	species	blood/plasma/ serum	measured
95		2050-68-2	PCB15	rat	blood	0.48
96		33284-52-5	PCB80	rat	blood	0.78
97		37680-73-2	PCB101	rat	blood	0.78
98		35065-27-1	PCB153	rat	blood	1.08
99		13551-89-8	ftuoromisonidazole	rat	blood	0.28
100		123-31-9	hydroquinone	rat	blood	-0.12
101		65873	bisphenolA	rat	blood	0.16
102		51333-22-3	budesonide	human	plasma	0.94
103		439-14-5	diazepam	rat	plasma	0.65
104		1622-62-4	ftunitrazepam	rat	plasma	0.57
105		846-49-1	lorazepam	rat	plasma	1.08
106		129-00-0	pyrene	rat	plasma	0.37
107		33671-46-4	clotiazepam	rat	blood	1.04
108		123-31-9	hydroquinone	rat	blood	-0.16
109		146-22-5	nitrazepam	rat	blood	0.26
110		51333-22-3	budesonide	rat	plasma	0.94
111		486-56-6	cotinine	rat	plasma	-0.18
112		439-14-5	diazepam	rat	plasma	0.52
113		846-49-1	lorazepam	rat	plasma	0.44
114		938-73-8	ethenzamide	rat	plasma	-0.03
115		129-00-0	pyrene	rat	plasma	0.35
116	**muscle**	33284-52-5	PCB 80	rat	blood	0
117		2051-62-9	PCB 3	rat	blood	0
118		2050-68-2	PCB 15	rat	blood	0.3
119		37680-73-2	PCB 101	rat	blood	0
120		35065-27-1	PCB 153	rat	blood	0.6

S. No	Tissue	CAS	Chemical	species	blood/plasma/ serum	measured
121		65936	p,p'-dichlorodiphenyl sulfone	rat	blood	0.58
122		15569-85-4	cotinine	rat	plasma	-0.05
123		439-14-5	diazepam	rat	plasma	0.15
124		938-73-8	ethenzamide	rat	plasma	-0.09
125		129-00-0	pyrene	rat	plasma	0.2
126	**skin**	35065-27-1	PCB 153	rat	blood	1.48
127		37680-73-2	PCB 101	rat	blood	0.85
128		33284-52-5	PCB 80	rat	blood	0.85
129		2050-68-2	PCB 15	rat	blood	1
130		2051-62-9	PCB 3	rat	blood	1
131		124-18-5	decane	rat	blood	0.68
132		65936	p,p'-dichlorodiphenyl sulfone	rat	blood	1.21
133		439-14-5	diazepam	rat	plasma	0.54
134		938-73-8	ethenzamide	rat	plasma	0.02
135		33671-46-4	clotiazepam	rabbit	plasma	0.15
136		439-14-5	diazepam	rabbit	plasma	0.2
137		146-22-5	nitrazepam	rabbit	plasma	0.2

[a] Data Source: [12]

Table S 10: List of experimental Milk-water partition coefficient and predicted values of Milk-water partitioning. [a]

S. No	Chemical	Exp Kmil_water	Predicted Kmil_water
1	Methyl acetate	-0.09	0
2	Ethyl acetate	-0.03	0.08
3	n-Propyl acetate	0.1	0.27

S. No	Chemical	Exp Kmil_water	Predicted Kmil_water
4	n-Butyl acetate	0.39	0.62
5	n-Pentyl acetate	0.93	1.09
6	Ethanol	-0.04	-0.03
7	Propan-2-ol	-0.04	-0.02
8	Butan-1-ol	-0.01	0.04
9	Pentan-1-ol	0.05	0.16
10	Heptan-1-ol	0.58	0.78
11	Octan-1-ol	1.09	1.23
12	Nonan-1-ol	1.66	1.77
13	n-Pentane	2.33	1.99
14	n-Hexane	2.91	2.55
15	n-Heptane	3.58	3.12
16	n-Octane	4.13	3.73
17	n-Nonane	4.73	4.3
18	n-Decane	5.34	4.88
19	Dichloromethane	0	0
20	Trichloromethane	0.73	0.81
21	Tetrachloromethane	1.88	1.63
22	1,1,2,2-Tetrachloroethane	1.24	1.23
23	1-Chloropentane	2.08	1.84
24	1-Chloroheptane	3.17	2.95
25	1-Chlorooctane	3.89	3.56
26	Butanal	-0.04	0.04
27	Hexanal	0.65	0.45
28	Heptanal	1.13	0.87
29	Octanal	1.67	1.39

S. No	Chemical	Exp Kmil_water	Predicted Kmil_water
30	Nonanal	2.08	1.93
31	1-Hexene	2.21	2.08
32	1-Heptene	2.7	2.65
33	1-Octene	3.29	3.22
34	1-Nonene	3.94	3.79
35	1-Decene	4.49	4.36
36	Benzene	0.93	0.99
37	Toluene	1.41	1.51
38	Ethylbenzene	1.9	2.03
39	n-Propylbenzene	2.44	2.6
40	n-Butylbenzene	2.89	3.15
41	Chlorobenzene	1.62	1.65
42	1,2-Dichlorobenzene	2.2	2.22
43	1,3-Dichlorobenzene	2.34	2.33
44	1,2,4-Trichlorobenzene	2.86	2.9
45	Di-n-propyl ether	0.77	0.8
46	Di-n-butyl ether	1.84	1.84
47	Dipentyl ether	2.88	3.01
48	2-Butanone	-0.04	0
49	2-Pentanone	0.03	0.09
50	2-Hexanone	0.19	0.29
51	2-Heptanone	0.58	0.64
52	2-Octanone	1.13	1.11
53	2-Nonanone	1.69	1.65
54	Nitromethane	-0.02	-0.02
55	Nitroethane	0.02	0.01

S. No	Chemical	Exp Kmil_water	Predicted Kmil_water
56	1-Nitropropane	0.11	0.12
57	1-Nitrobutane	0.36	0.41
58	1-Nitrohexane	1.35	1.29
59	Nitrobenzene	0.74	0.75
60	2-Nitrotoluene	1.18	1.23
61	2,6-Dinitrotoluene	1.04	1.01
62	2,4-Dinitrotoluene	1.01	1.01
63	1-Chloro-4-nitrobenzene	1.24	1.31
64	Anthracene	3.53	3.61
65	Phenanthrene	3.5	3.46
66	Fluoranthene	3.9	4.3
67	Pyrene	3.98	4.06
68	Fluorene	3.08	3.41
69	Cyclopentane	2.29	1.63
70	Cyclohexane	2.69	2.26
71	Cycloheptane	3.41	2.85
72	Cyclooctane	4.11	3.46
73	Cyclohexene	2.01	1.66
74	Methylcyclohexane	3.17	2.82
75	Methylcyclopentane	3.1	2.2
76	Ethyl tert-butyl ether	0.3	0.4
77	Ethyl tert-pentyl ether	0.74	0.8
78	Diisopropyl ether	0.08	0.44
79	Halothane	1.07	1.03
80	Enflurane	0.75	0.88
81	Isoflurane	0.83	0.94

S. No	Chemical	Exp Kmil_water	Predicted Kmil_water
82	Methoxyflurane	0.93	1
83	Hexafluorobenzene	1.11	1.48
84	Methylpentafluorobenzene	1.79	1.94
85	Pentafluorobenzene	0.97	1.29
86	2-Ethyl-1-hexanol	0.94	1.26
87	3-Ethyl-3-hexanol	0.59	0.97
88	4-Ethyl-3-hexanol	0.77	0.99
89	3-Ethyl-3-pentanol	0.18	0.55
90	Tetrachloroethene	2.26	2.09
91	Trichloroethene	1.51	1.34
92	3-Chlorophenol	0.67	0.87
93	4-Chlorophenol	0.57	0.73
94	4-Ethylpyridine	0.16	0.26
95	2-Chloroaniline	0.81	0.82
96	Acenaphthene	2.65	3
97	4-Nitroanisole	1	0.92
98	1,4-Dimethoxybenzene	0.81	0.83
99	Ethyl benzoate	1.25	1.36
100	Indole	0.82	0.98
101	Biphenyl	2.82	2.89
102	1-Naphthol	1.28	1.26
103	2,2,4-Trimethylpentane	3.02	3.73
104	4-Bromophenol	0.76	0.9
105	4-Iodoaniline	0.92	0.92
106	N,N-Diethylaniline	1.32	2.08
107	4-n-Propylphenol	1.08	1.19

S. No	Chemical	Exp Kmil_water	Predicted Kmil_water
108	4-Iodophenol	1.08	1.25

[a] Data Source: [12]

Table S 11: Training set for $\boldsymbol{logK_{pw}}$

S. No	***Chemicals***	***CAS-RN***	***SMILES***	$\boldsymbol{logK_{pw}}$	$\boldsymbol{logK_{ow}}$	$\boldsymbol{logK_{aw}}$
1	*Cyclooctane*	*292-64-8*	*C1CCCCCCC1*	*2.88*	*4.45*	*1.18*
2	*Cycloheptane*	*291-64-5*	*C1CCCCCC1*	*2.25*	*4.00*	*0.58*
3	*Non-1-ene*	*124-11-8*	*CCCCCCCC=C*	*3.53*	*5.15*	*1.51*
4	*Hept-1-yne*	*628-71-7*	*CCCCCC#C*	*1.69*	*3.44*	*0.46*
5	*1-Chlorooctane*	*111-85-3*	*CCCCCCCCCl*	*2.91*	*3.64*	*0.19*
6	*Di-n-butyl ether*	*142-96-1*	*CCCCOCCCC*	*1.62*	*3.21*	*-0.61*
7	*Octan-2-one*	*111-13-7*	*CCCCCCC(=O)C*	*1.46*	*2.37*	*-2.11*
8	*Nonan-2-one*	*821-55-6*	*CCCCCCCC(=O)C*	*1.67*	*3.14*	*-1.82*
9	*Decan-2-one*	*693-54-9*	*CCCCCCCCC(=O)C*	*2.04*	*3.73*	*-1.91*
10	*1-Nitrohexane*	*646-14-0*	*CCCCCCN(=O)=O*	*1.34*	*2.70*	*-2.03*
11	*Hexan-1-ol*	*111-27-3*	*CCCCCCO*	*0.55*	*2.03*	*-3.16*
12	*Octan-1-ol*	*111-87-5*	*CCCCCCCCO*	*1.49*	*3.00*	*-3.00*
13	*Nonan-1-ol*	*143-08-8*	*CCCCCCCCCO*	*1.98*	*3.77*	*-2.90*
14	*4-Ethyl-3-hexanol*	*19780-44-0*	*CCC(C(CC)O)CC*	*0.75*	*2.78*	*-2.85*

S. No	Chemicals	CAS-RN	SMILES	$log K_{pw}$	$log K_{ow}$	$log K_{aw}$
15	*n-Propylbenzene*	*103-65-1*	*CCCc1ccccc1*	*1.97*	*3.69*	*-0.37*
16	*n-Pentylbenzene*	*538-68-1*	*CCCCCc1ccccc1*	*2.74*	*4.90*	*0.02*
17	*n-Hexylbenzene*	*1077-16-3*	*CCCCCCc1ccccc1*	*3.22*	*5.52*	*0.07*
18	*1,2,4-Trimethylbenzene*	*95-63-6*	*Cc1ccc(c(c1)C)C*	*1.89*	*3.63*	*-0.60*
19	*1,2,3,4-Tetrachlorobenzene*	*634-66-2*	*Clc1c(Cl)ccc(c1Cl)Cl*	*2.83*	*4.60*	*-1.51*
20	*1,2,4,5-Tetrachlorobenzene*	*95-94-3*	*Clc1cc(Cl)c(cc1Cl)Cl*	*2.97*	*4.64*	*-1.39*
21	*Pentachlorobenzene*	*608-93-5*	*Clc1cc(Cl)c(c(c1Cl)Cl)Cl*	*3.38*	*5.17*	*-1.54*
22	*1,4-Dibromobenzene*	*106-37-6*	*Brc1ccc(cc1)Br*	*2.23*	*3.79*	*-1.44*
23	*Dibenzothiophene*	*132-65-0*	*c1ccc2c(c1)sc1c2cccc1*	*3.43*	*4.38*	*-2.86*
24	*Pyrene*	*129-00-0*	*c1cc2ccc3c4c2c(c1)ccc4ccc3*	*3.72*	*4.88*	*-3.31*
25	*Chrysene*	*218-01-9*	*c1ccc2c(c1)c1ccc3c(c1cc2)cccc3*	*4.47*	*5.81*	*-3.67*
26	*Benzo[a]pyrene*	*50-32-8*	*c1ccc2c(c1)c1ccc3c4c1c(c2)ccc4ccc3*	*4.93*	*6.13*	*-4.73*
27	*4-Nitroanisole*	*100-17-4*	*COc1ccc(cc1)N(=O)=O*	*1.38*	*2.03*	*-4.21*
28	*Valerophenone*	*1009-14-9*	*CCCCC(=O)c1ccccc1*	*1.75*	*3.29*	*-2.98*
29	*Benzophenone*	*119-61-9*	*O=C(c1ccccc1)c1ccccc1*	*2.00*	*3.18*	*-4.46*
30	*di-n-propyl phthalate*	*131-16-8*	*CCCOC(=O)c1ccccc1C(=O)OCCC*	*1.92*	*3.27*	*-4.78*
31	*2-Nitrotoluene*	*88-72-2*	*O=N(=O)c1ccccc1C*	*1.30*	*2.30*	*-3.29*
32	*1-Nitronaphthalene*	*86-57-7*	*O=N(=O)c1cccc2c1cccc2*	*2.16*	*3.19*	*-4.14*

S. No	Chemicals	CAS-RN	SMILES	$logK_{pw}$	$logK_{ow}$	$logK_{aw}$
33	*4-n-Propylphenol*	*645-56-7*	*CCCc1ccc(cc1)O*	*1.66*	*3.20*	*-4.33*
34	*4-Fluorophenol*	*371-41-5*	*Oc1ccc(cc1)F*	*1.14*	*1.77*	*-4.54*
35	*4-Chlorophenol*	*106-48-9*	*Oc1ccc(cc1)Cl*	*1.50*	*2.39*	*-4.59*
36	*4-Bromophenol*	*106-41-2*	*Oc1ccc(cc1)Br*	*1.66*	*2.59*	*-5.21*
37	*4-Iodophenol*	*540-38-5*	*Oc1ccc(cc1)I*	*1.89*	*2.91*	*-5.41*
38	*2-Phenylphenol*	*90-43-7*	*Oc1ccccc1c1ccccc1*	*2.00*	*3.09*	*-4.37*
39	*4-Chloroaniline*	*106-47-8*	*Nc1ccc(cc1)Cl*	*1.19*	*1.83*	*-4.32*
40	*4-Iodoaniline*	*540-37-4*	*Nc1ccc(cc1)I*	*1.66*	*2.34*	*-5.14*
41	*4-Nitroaniline*	*100-01-6*	*Nc1ccc(cc1)N(=O)=O*	*0.84*	*1.39*	*-7.29*
42	*N,N-Diethylaniline*	*91-66-7*	*CCN(c1ccccc1)CC*	*1.56*	*3.31*	*-2.11*
43	*Indole*	*120-72-9*	*c1ccc2c(c1)[nH]cc2*	*1.69*	*2.14*	*-4.67*

Table S 12: Validation set for $\boldsymbol{log\ K_{pw}}$

S. No	Chemicals	CAS-RN	SMILES	$logK_{pw}$	$logK_{ow}$	$logK_{aw}$
1	*n-Nonane*	*111-84-2*	*CCCCCCCCC*	*3.77*	*5.65*	*2.14*
2	*Tri-n-butyl phosphate*	*126-73-8*	*CCCCOP(=O)(OCCCC)OCCCC*	*1.76*	*4.00*	*-4.24*
3	*1,2-Dichlorobenzene*	*95-50-1*	*Clc1ccccc1Cl*	*2.03*	*3.43*	*-1.11*
4	*Phenanthrene*	*85-01-8*	*c1ccc2c(c1)c1ccccc1cc2*	*3.20*	*4.46*	*-2.76*

5	*Ethyl benzoate*	*93-89-0*	*CCOC(=O)c1ccccc1*	*1.27*	*2.64*	*-2.52*
6	*2,4-Dinitrotoluene*	*121-14-2*	*O=N(=O)c1ccc(c(c1)N(=O)=O)C*	*1.25*	*1.98*	*-5.66*
7	*3-Chlorophenol*	*108-43-0*	*Oc1cccc(c1)Cl*	*1.56*	*2.50*	*-4.85*
8	*Diazepam*	*439-14-5*	*Clc1ccc2c(c1)C(=NCC(=O)N2C)c1ccccc1*	*2.06*	*2.82*	*-8.59*

Table S 13: Training set for $log\ K_{BSA}$

S. No	***Chemicals***	***CAS-RN***	***SMILES***	$\log K_{BSA}$	$\log K_{ow}$	$\log K_{aw}$
1	n-heptane	142-82-5	CCCCCCC	3.59	4.66	1.92
2	n-octane	111-65-9	CCCCCCCC	4.01	5.18	2.13
3	cyclohexane	110-82-7	C1CCCCC1	2.01	3.44	0.80
4	cycloheptane	291-64-5	C1CCCCCC1	2.52	4.00	0.59
5	cyclooctane	292-64-8	C1CCCCCCC1	2.98	4.45	1.18
6	1-nonene	124-11-8	CCCCCCCC=C	4.22	5.15	1.52
7	1-heptyne	628-71-7	CCCCCC#C	2.49	3.44	0.47
8	tetrachlorometha ne	56-23-5	ClC(Cl)(Cl)Cl	1.77	2.83	0.06
9	trichloroethene	79-01-6	ClC=C(Cl)Cl	1.88	2.42	-0.39
10	tetrachloroethen e	127-18-4	ClC(=C(Cl)Cl)Cl	2.40	3.40	-0.13
11	tribromomethane	75-25-2	BrC(Br)Br	1.95	2.40	-1.65

S. No	*Chemicals*	*CAS-RN*	*SMILES*	$logK_{BSA}$	$logK_{ow}$	$logK_{aw}$
12	g-hexachlorocyclohexane	58-89-9	Cl[C@@H]1[C@H](Cl)[C@@H](Cl)[C@@H]([C@@H]([C@H]1Cl)Cl)Cl	2.46	4.14	-3.67
13	isoflurane	26675-46-7	FC(OC(C(F)(F)F)Cl)F	1.58	2.06	0.08
14	enflurane	13838-16-9	FC(OC(C(Cl)F)(F)F)F	1.59	2.10	0.03
15	methoxyflurane	76-38-0	COC(C(Cl)Cl)(F)F	1.77	2.21	-0.81
16	di-n-butyl ether	142-96-1	CCCCOCCCC	2.01	3.21	-0.60
17	2-octanone	111-13-7	CCCCCCC(=O)C	2.09	2.37	-2.11
18	2-nonanone	821-55-6	CCCCCCCC(=O)C	2.48	3.14	-1.82
19	2-decanone	693-54-9	CCCCCCCCC(=O)C	2.88	3.73	-1.91
20	1-nitrooctane	629-37-8	CCCCCCCCN(=O)=O	3.38	3.57	-1.67
21	benzene	71-43-2	c1ccccc1	1.58	2.13	-0.64
22	toluene	108-88-3	Cc1ccccc1	2.26	2.73	-0.56
23	ethylbenzene	100-41-4	CCc1ccccc1	2.70	3.15	-0.48
24	n-propylbenzene	103-65-1	CCCc1ccccc1	2.95	3.69	-0.36
25	styrene	100-42-5	C=Cc1ccccc1	2.76	2.95	-0.94
26	chlorobenzene	108-90-7	Clc1ccccc1	2.32	2.84	-0.89

S. No	Chemicals	CAS-RN	SMILES	$logK_{BSA}$	$logK_{ow}$	$logK_{aw}$
27	1,2,4-trimethylbenzene	95-63-6	Cc1ccc(c(c1)C)C	3.35	3.63	-0.59
28	1,4-dibromobenzene	106-37-6	Brc1ccc(cc1)Br	3.97	3.79	-1.43
29	1,2,4-trichlorobenzene	120-82-1	Clc1ccc(c(c1)Cl)Cl	3.60	4.02	-1.23
30	1,2,3,4-tetrachlorobenzene	634-66-2	Clc1c(Cl)ccc(c1Cl)Cl	4.21	4.60	-1.50
31	hexafluorobenzene	392-56-3	Fc1c(F)c(F)c(c(c1F)F)F	1.55	2.55	0.35
32	methylpentafluorobenzene	771-56-2	Fc1c(C)c(F)c(c(c1F)F)F	2.32	3.07	0.17
33	indene	95-13-6	c1ccc2c(c1)C=CC2	2.92	2.92	-1.65
34	naphthalene	91-20-3	c1ccc2c(c1)cccc2	3.56	3.30	-1.74
35	dibenzofuran	132-64-9	c1ccc2c(c1)oc1c2cccc1	3.79	4.12	-2.05
36	dibenzothiophene	132-65-0	c1ccc2c(c1)sc1c2cccc1	4.16	4.38	-2.85
37	fluoranthene	206-44-0	c1ccc2c(c1)c1cccc3c1c2ccc3	4.28	5.16	-3.43
38	pyrene	129-00-0	c1cc2ccc3c4c2c(c1)ccc4ccc3	4.76	4.88	-3.30

S. No	Chemicals	CAS-RN	SMILES	$logK_{BSA}$	$logK_{ow}$	$logK_{aw}$
39	chrysene	218-01-9	c1ccc2c(c1)c1ccc3c(c1cc2)cccc3	4.46	5.81	-3.66
40	benzo[b]fluoranthene	205-99-2	c1ccc2c(c1)c1cc3ccccc3c3c1c2ccc3	4.42	5.78	-4.56
41	benzo[ghi]perylene	191-24-2	c1cc2ccc3c4c2c(c1)c1cccc2c1c4c(cc3)cc2	4.76	6.76	-6.59
42	anisole	100-66-3	COc1ccccc1	2.16	2.11	-1.70
43	valerophenone	1009-14-9	CCCCC(=O)c1ccccc1	2.70	3.29	-2.98
44	benzophenone	119-61-9	O=C(c1ccccc1)c1ccccc1	2.62	3.18	-4.46
45	di-n-propyl phthalate	131-16-8	CCCOC(=O)c1ccccc1C(=O)OCCC	2.84	3.27	-4.77
46	2,4-dinitrotoluene	121-14-2	O=N(=O)c1ccc(c(c1)N(=O)=O)C	1.73	1.98	-5.65
47	1-nitronaphthalene	86-57-7	O=N(=O)c1cccc2c1cccc2	3.17	3.19	-4.13
48	4-nitroanisole	100-17-4	COc1ccc(cc1)N(=O)=O	2.48	2.03	-4.67
49	N,N-diethylaniline	91-66-7	CCN(c1ccccc1)CC	2.27	3.31	-2.10
50	1-hexanol	111-27-3	CCCCCCO	1.64	2.03	-3.15

S. No	Chemicals	CAS-RN	SMILES	$logK_{BSA}$	$logK_{ow}$	$logK_{aw}$
51	1-octanol	111-87-5	CCCCCCCCO	2.74	3.00	-2.99
52	1-nonanol	143-08-8	CCCCCCCCCO	3.10	3.77	-2.89
53	4-ethyl-3-hexanol	19780-44-0	CCC(C(CC)O)CC	1.48	2.78	-2.85
54	4-n-propylphenol	645-56-7	CCCc1ccc(cc1)O	2.59	3.20	-4.32
55	2-phenylphenol	90-43-7	Oc1ccccc1c1ccccc1	2.62	3.09	-4.36
56	3-chlorophenol	108-43-0	Oc1cccc(c1)Cl	2.35	2.50	-4.84
57	4-bromophenol	106-41-2	Oc1ccc(cc1)Br	2.81	2.59	-5.20
58	4-iodophenol	540-38-5	Oc1ccc(cc1)I	3.41	2.91	-5.41
59	bisphenol A	80-05-7	CC(c1ccc(cc1)O)(c1ccc(cc1)O)C	2.88	3.32	-10.56
60	4-nitroaniline	100-01-6	Nc1ccc(cc1)N(=O)=O	1.69	1.39	-7.28
61	2-chloroaniline	95-51-2	Nc1ccccc1Cl	1.95	1.90	-3.65
62	4-aminobiphenyl	92-67-1	Nc1ccc(cc1)c1ccccc1	2.55	2.86	-5.83
63	indole	120-72-9	c1ccc2c(c1)[nH]cc2	2.25	2.14	-4.66
64	atrazine	1912-24-9	CCNc1nc(NC(C)C)nc(n1)Cl	1.77	2.61	-7.01

S. No	Chemicals	CAS-RN	SMILES	$logK_{BSA}$	$logK_{ow}$	$logK_{aw}$
65	diazepam	439-14-5	Clc1ccc2c(c1)C(=NCC(=O)N2C)c1ccccc1	2.68	2.82	-8.59
66	estrone	53-16-7	Oc1ccc2c(c1)CC[C@@H]1[C@@H]2CC[C@]2([C@H]1CCC2=O)C	2.69	3.13	-10.43
67	endosulfan a	115-29-7	O=S1OCC2C(CO1)C1(C(C2(Cl)C(=C1Cl)Cl)(Cl)Cl)Cl	3.24	3.83	-2.57

Table S 14: Validation set for $log\ K_{BSA}$

S. No	Chemicals	CAS-RN	SMILES	$logK_{BSA}$	$logK_{ow}$	$logK_{aw}$
1	*n-hexane*	*110-54-3*	*CCCCCC*	*3.09*	*3.90*	*1.88*
2	*n-nonane*	*111-84-2*	*CCCCCCCCC*	*4.45*	*5.65*	*2.15*
3	*1-chlorooctane*	*111-85-3*	*CCCCCCCCCl*	*3.85*	*4.64*	*0.20*
4	*halothane*	*151-67-7*	*ClC(C(F)(F)F)Br*	*1.62*	*2.30*	*-0.07*
5	*di-n-pentyl ether*	*693-65-2*	*CCCCCOCCCCC*	*3.00*	*4.28*	*-0.44*
6	*tri-n-butyl phosphate*	*126-73-8*	*CCCCOP(=O)(OCCCC)OCCCC*	*2.47*	*4.00*	*-4.23*
7	*1,2-dichlorobenzene*	*95-50-1*	*Clc1ccccc1Cl*	*3.03*	*3.43*	*-1.10*
8	*phenanthrene*	*85-01-8*	*c1ccc2c(c1)c1ccccc1cc2*	*4.15*	*4.46*	*-2.75*

S. No	Chemicals	CAS-RN	SMILES	$logK_{BSA}$	$logK_{ow}$	$logK_{aw}$
9	*2-nitrotoluene*	*88-72-2*	*O=N(=O)c1ccccc1C*	*2.12*	*2.30*	*-3.28*
10	*1-heptanol*	*111-70-6*	*CCCCCCCO*	*2.18*	*2.62*	*-3.11*
11	*4-chlorobenzyl alcohol*	*873-76-7*	*OCc1ccc(cc1)Cl*	*2.10*	*1.96*	*-5.02*
12	*4-fluorophenol*	*371-41-5*	*Oc1ccc(cc1)F*	*1.57*	*1.77*	*-4.53*
13	*4-chlorophenol*	*106-48-9*	*Oc1ccc(cc1)Cl*	*2.43*	*2.39*	*-4.58*
14	*4-iodoaniline*	*540-37-4*	*Nc1ccc(cc1)I*	*2.95*	*2.34*	*-5.14*
15	*carbazole*	*86-74-8*	*c1ccc2c(c1)[nH]c1c2cccc1*	*3.52*	*3.72*	*-5.32*
16	*metolachlor*	*51218-45-2*	*COCC(N(c1c(C)cccc1CC)C(=O)CCl)C*	*1.74*	*2.90*	*-6.43*

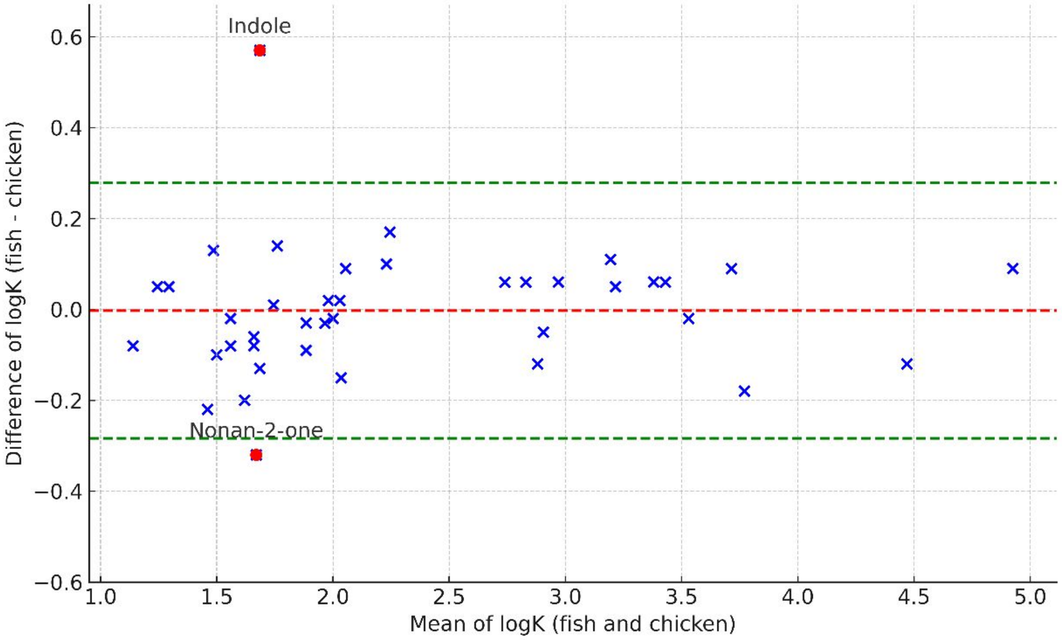

Figure S 1 Bland-Altman Plot illustrating the agreement between fish and chicken protein-water partition coefficients. The plot displays the differences between the log K values for fish and chicken proteins against their average. The central red dashed line represents the mean difference, while the upper and lower green dashed lines define the limits of agreement (±1.96 standard deviations from the mean difference). Two outliers, nonan-2-one and indole, are marked in red and annotated, indicating instances where the agreement between fish and chicken protein measurements significantly deviates from the majority of data points.

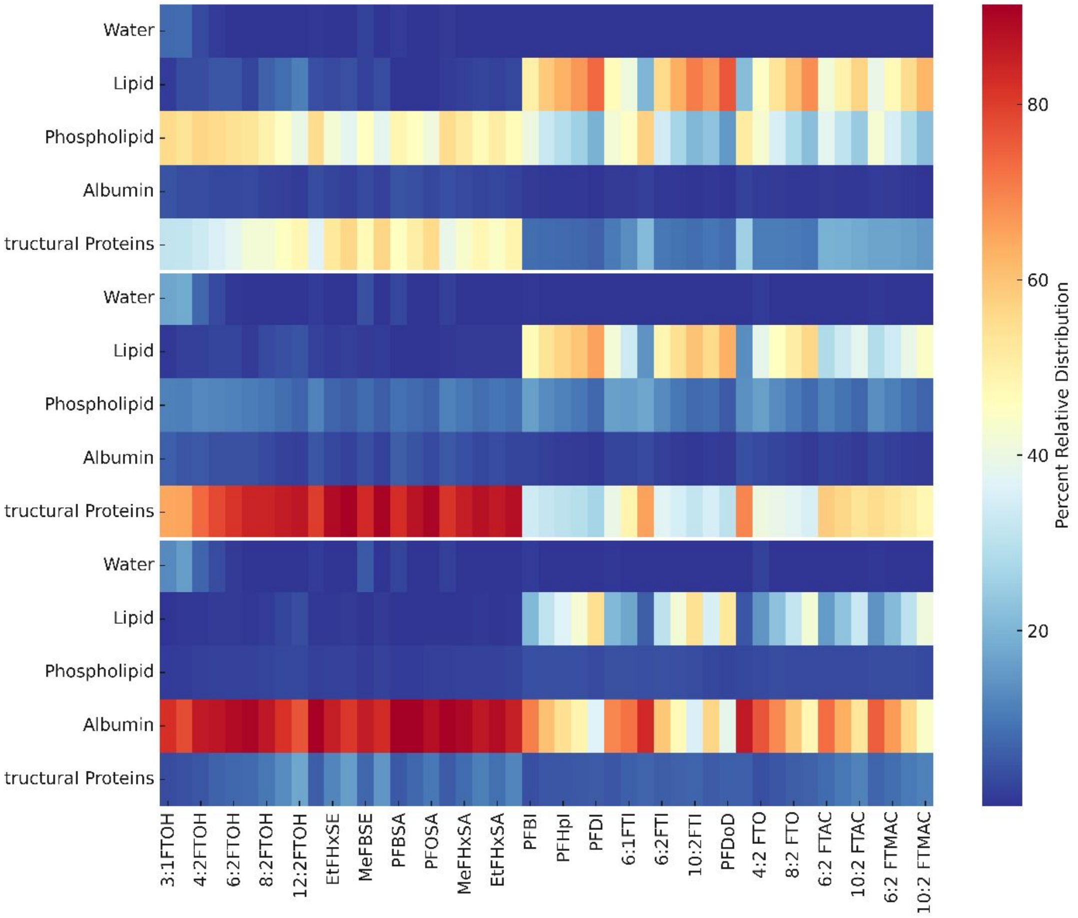

Figure S 2: Relative distribution of neutral per- and polyfluoroalkyl substances (PFAS) across various phases including water, albumin, structural proteins, lipids, and phospholipids in mammalian tissues/fluids such as plasma, muscle, and liver. These distributions are obtained through multiphase partitioning modeling based on predicted partition coefficients via pp-LFER

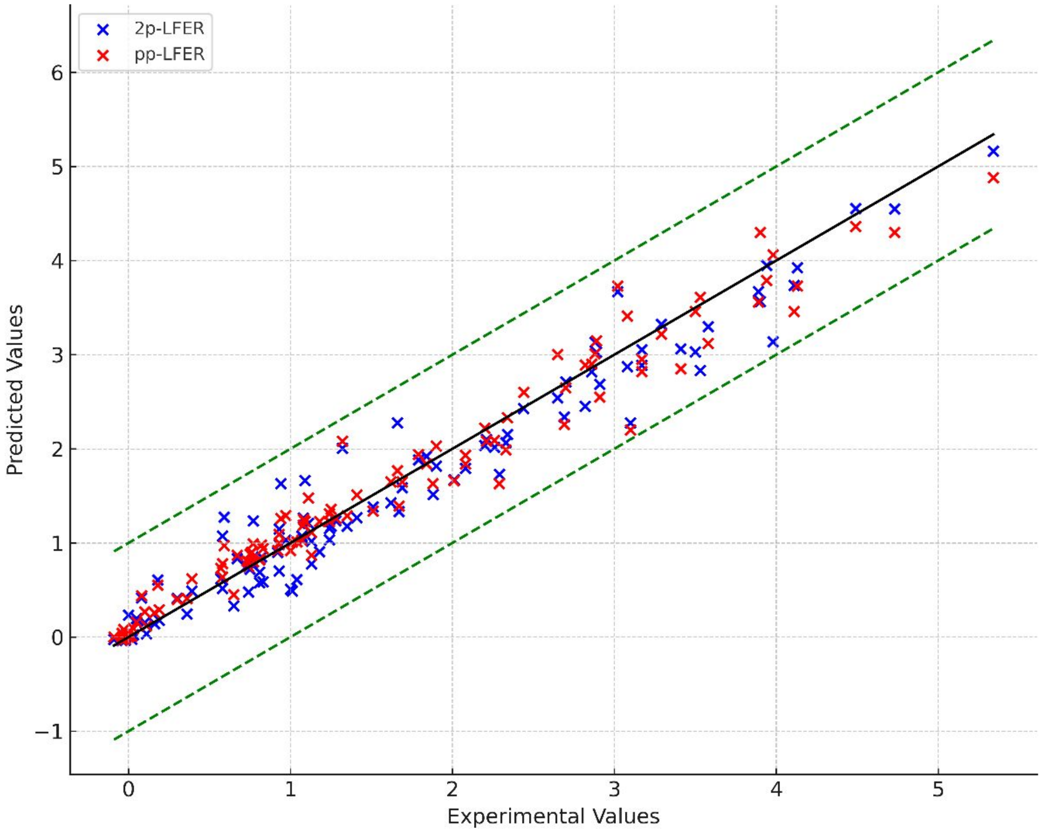

Figure S 3**:** The agreement between experimental milk-to-water partition coefficients and predicted values using 2p-LFER (blue points) and pp-LFER (red points). The black line represents the unity line where predicted values equal experimental values. The green dashed lines are positioned one log unit above and below the unity line, illustrating the deviation of predicted values from the experimental data. This visualization aids in comparing the accuracy of 2p-LFER and pp-LFER models in predicting partition coefficients.

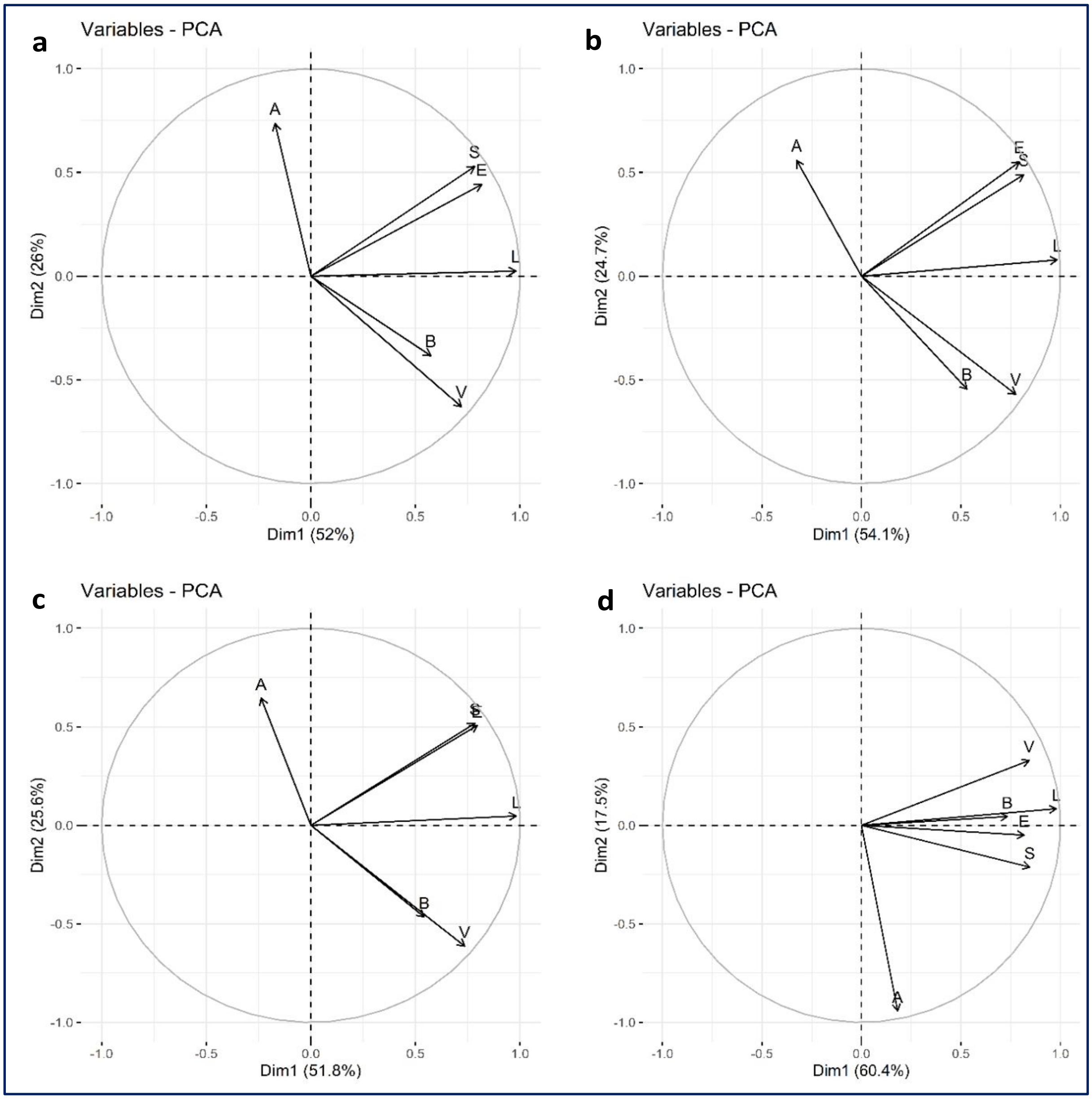

Figure S 4:. PCA plots performed on Abraham solute descriptors (ASDs) of $logK_{ch}$ (**a**), $logK_{fish}$ (**b**), $logK_{pw}$ (**c**), and $logK_{BSA}$ (**d**) indicating the values of first two dimensions. Further strengthening our hypothesis that only two dimensions are enough to scout maximum information coded by ASDs

Section 1: Cross validation of all models

Here we will present the results of cross validation analysis for each model. Three different types of cross-validation tests such as leave-one-out, k-fold (k=10), and bootstrapping with 1000 resamples were performed on training and validation sets so that the internal validation, robustness, and predictive capability of each model could be assessed.

a. *Cross-validation of $logK_{ch}$ 2p-LFER Model (n = 46)*

Test	RMSE	R^2	MAE
Leave-One-Out Cross-Validation	0.337	0.863	0.255
10-fold cross-validation	0.301	0.884	0.243
Bootstrapped resampling (1000 reps)	0.343	0.866	0.265

b. *Cross-validation of $logK_{fish}$ 2p-LFER Model (n = 45)*

Test	RMSE	R^2	MAE
Leave-One-Out Cross-Validation	0.364	0.852	0.276
10-fold cross-validation	0.346	0.884	0.280
Bootstrapped resampling (1000 reps)	0.371	0.850	0.287

c. *Cross-validation of $logK_{pw}$ 2p-LFER Model (n = 51)*

Test	RMSE	R^2	MAE
Leave-One-Out Cross-Validation	0.344	0.862	0.264
10-fold cross-validation	0.316	0.881	0.266
Bootstrapped resampling (1000 reps)	0.353	0.857	0.273

d. Cross-validation of $logK_{BSA}$ 2p-LFER Model (n = 83)

Test	RMSE	R^2	MAE
Leave-One-Out Cross-Validation	0.433	0.747	0.337
10-fold cross-validation	0.429	0.765	0.340
Bootstrapped resampling (1000 reps)	0.438	0.753	0.344

Section 2. R codes

```
# Load necessary libraries
library(caret)
library(corrplot)
library(ggplot2)
library(reshape2)
library(FactoMineR)
library(factoextra)
library(psych)
library(dplyr)
library(magrittr)
library(Metrics)

# 1. Function for Data Import and Preprocessing
import_and_preprocess_data <- function(url) {
  data <- read.csv(url)
  summary(data)
  mydata <- data[, c(5:13)]  # Adjust the column indices if necessary
  return(list(data = data, mydata = mydata))
}

# 2. Function for Correlation Analysis and Plotting
perform_correlation_analysis <- function(mydata) {
  cormat <- round(cor(mydata), 2)

  # Get the upper triangle of the correlation matrix
  get_upper_tri <- function(cormat){
    cormat[lower.tri(cormat)] <- NA
    return(cormat)
  }

  upper_tri <- get_upper_tri(cormat)
```

```
  melted_cormat <- melt(upper_tri, na.rm = TRUE)

  # Create the heatmap
  ggheatmap <- ggplot(melted_cormat, aes(Var2, Var1, fill = value)) +
    geom_tile(color = "white") +
    scale_fill_gradient2(low = "blue", high = "red", mid = "white",
                         midpoint = 0, limit = c(-1, 1), space = "Lab",
                         name = "Pearson\nCorrelation") +
    theme_minimal() +
    theme(
      axis.text.x = element_text(angle = 45, vjust = 1, size = 12, hjust = 1, colour = "blue"),
      axis.text.y = element_text(vjust = 1, size = 12, hjust = 1, colour = "blue")
    ) +
    coord_fixed()

  ggheatmap <- ggheatmap +
    geom_text(aes(Var2, Var1, label = value), color = "black", size = 4) +
    theme(
      axis.title.x = element_blank(),
      axis.title.y = element_blank(),
      panel.grid.major = element_blank(),
      panel.border = element_blank(),
      panel.background = element_blank(),
      axis.ticks = element_blank(),
      legend.justification = c(1, 0),
      legend.position = c(0.6, 0.7),
      legend.direction = "horizontal"
    ) +
    guides(fill = guide_colorbar(
      barwidth = 7, barheight = 1,
      title.position = "top", title.hjust = 0.5
    ))

  print(ggheatmap)
}

# 3. Function for PCA Analysis
perform_pca_analysis <- function(data, variables) {
  res.pca <- prcomp(data[, variables], scale = TRUE)
  print(res.pca)

  var <- get_pca_var(res.pca)

  fviz_pca_var(res.pca, col.var = "black",
               labelsize = 4,
               pointsize = 2,
```

```
             arrowsize = 0.6,
             font.x = 12, font.y = 12)

  corrplot(var$cos2, is.corr = FALSE, tl.cex = 1.2, cl.cex = 0.8, tl.col = "red")
}

# 4. Function for Regression Analysis
run_regression_analysis <- function(formula, data) {
  model <- lm(formula, data = data)
  summary_model <- summary(model)
  print(summary_model)
  return(model)
}

# 5. Function for Cross-Validation
perform_cross_validation <- function(formula, data, dependent_var) {
  set.seed(123)

  # Split the data into training and testing sets
  training.samples <- data[[dependent_var]] %>% createDataPartition(p = 0.8, list = FALSE)
  train.data <- data[training.samples, ]
  test.data <- data[-training.samples, ]

  # Train the model
  model <- lm(formula, data = train.data)

  # Make predictions
  predictions <- predict(model, test.data)

  # Model performance metrics
  results <- data.frame(
    R2 = R2(predictions, test.data[[dependent_var]]),
    RMSE = RMSE(predictions, test.data[[dependent_var]]),
    MAE = MAE(predictions, test.data[[dependent_var]])
  )
  print(results)

  # Normalized RMSE
  normalized_rmse <- RMSE(predictions, test.data[[dependent_var]]) /
mean(test.data[[dependent_var]])
  print(paste("Normalized RMSE:", normalized_rmse))

  # Leave One Out Cross Validation
  train.control_Loo <- trainControl(method = "LOOCV")
  model_Loo <- train(formula, data = data, method = "lm", trControl = train.control_Loo)
  print(model_Loo)
```

```
  # K-fold Cross Validation
  train.control_cv <- trainControl(method = "cv", number = 10)
  model_cv <- train(formula, data = data, method = "lm", trControl = train.control_cv)
  print(model_cv)

  # Bootstrapping Cross Validation
  train.control_boot <- trainControl(method = "boot", number = 1000)
  model_boot <- train(formula, data = data, method = "lm", trControl = train.control_boot)
  print(model_boot)
}

# 6. Functions for Predicting Values and Calculating RMSE
calculate_predicted_values <- function(df, formula_func, experimental_col) {
  df$predicted_values <- with(df, formula_func(logKow, logKaw))
  print(df)

  # Calculate RMSE
  calculated_rmse <- rmse(df[[experimental_col]], df$predicted_values)
  print(paste("RMSE:", calculated_rmse))
}

# Define the prediction formulas
predict_logKpw <- function(logKow, logKaw) {
  -1.080 + 0.851 * logKow - 0.092 * logKaw
}

predict_logKBSA <- function(logKow, logKaw) {
  0.788 * logKow - 0.053 * logKaw
}

# 7. Define datasets with URLs and dependent variables
datasets <- list(
  list(url = "https://zenodo.org/record/14062718/files/Table_S1.csv?download=1", dep_var =
"logKch"),
  list(url = "https://zenodo.org/record/14062718/files/Table_S2.csv?download=1", dep_var =
"logKfish"),
  list(url = "https://zenodo.org/record/14062718/files/Table_S3.csv?download=1", dep_var =
"logKBSA"),
  list(url = "https://zenodo.org/record/14062718/files/Table_S4.csv?download=1", dep_var =
"logKpw")
  # Include other tables as needed
)

variables_for_pca <- 5:10  # Adjust according to your data
```

```
# 8. Loop through datasets
for (dataset in datasets) {
  data_list <- import_and_preprocess_data(dataset$url)
  data <- data_list$data
  mydata <- data_list$mydata

  perform_correlation_analysis(mydata)
  perform_pca_analysis(data, variables = variables_for_pca)

  formula <- as.formula(paste(dataset$dep_var, "~ logKow + logKaw"))
  model <- run_regression_analysis(formula, data)
  perform_cross_validation(formula, data, dependent_var = dataset$dep_var)
}

# 9. Handle specific cases for Tables S7 and S8

# For Table_S7.csv (Ionizable PFAS Compounds)
df_S7 <- read.csv("https://zenodo.org/record/14062718/files/Table_S7.csv?download=1")

# Predict logKpw values and calculate RMSE
df_S7$predicted_logKpw <- with(df_S7, predict_logKpw(logKow, logKaw))
rmse_logKpw_S7 <- rmse(df_S7$experimental_logKpw, df_S7$predicted_logKpw)
print("Predictions and RMSE for logKpw (Table S7):")
print(df_S7[, c("logKow", "logKaw", "experimental_logKpw", "predicted_logKpw")])
print(paste("RMSE for logKpw (Table S7):", rmse_logKpw_S7))

# Predict logKBSA values and calculate RMSE
df_S7$predicted_logKBSA <- with(df_S7, predict_logKBSA(logKow, logKaw))
rmse_logKBSA_S7 <- rmse(df_S7$experimental_logKBSA, df_S7$predicted_logKBSA)
print("Predictions and RMSE for logKBSA (Table S7):")
print(df_S7[, c("logKow", "logKaw", "experimental_logKBSA", "predicted_logKBSA")])
print(paste("RMSE for logKBSA (Table S7):", rmse_logKBSA_S7))

# For Table_S8.csv (Neutral PFAS Compounds)
df_S8 <- read.csv("https://zenodo.org/record/14062718/files/Table_S8.csv?download=1")

# Predict logKpw values and calculate RMSE for Muscle ASM
df_S8$predicted_logKpw <- with(df_S8, predict_logKpw(logKow, logKaw))
rmse_logKpw_S8 <- rmse(df_S8$Muscle_ASM, df_S8$predicted_logKpw)
print("Predictions and RMSE for Muscle ASM (logKpw, Table S8):")
print(df_S8[, c("logKow", "logKaw", "Muscle_ASM", "predicted_logKpw")])
print(paste("RMSE for Muscle ASM (Table S8):", rmse_logKpw_S8))

# Predict logKBSA values and calculate RMSE for Albumin ASM
df_S8$predicted_logKBSA <- with(df_S8, predict_logKBSA(logKow, logKaw))
rmse_logKBSA_S8 <- rmse(df_S8$Albumin_ASM, df_S8$predicted_logKBSA)
```

```
print("Predictions and RMSE for Albumin ASM (logKBSA, Table S8):")
print(df_S8[, c("logKow", "logKaw", "Albumin_ASM", "predicted_logKBSA")])
print(paste("RMSE for Albumin ASM (Table S8):", rmse_logKBSA_S8))
```

References

(1) Endo, S.; Bauerfeind, J.; Goss, K. U. Partitioning of Neutral Organic Compounds to Structural Proteins. *Environ. Sci. Technol.* **2012**, *46*, 12697–12703. https://doi.org/10.1021/es303379y.

(2) Endo, S.; Goss, K. U. Serum Albumin Binding of Structurally Diverse Neutral Organic Compounds: Data and Models. *Chem. Res. Toxicol.* **2011**, *24*, 2293–2301. https://doi.org/10.1021/tx200431b.

(3) EPA, U. S. Estimation Programs Interface Suite™ for Microsoft® Windows. *United States Environ. Prot. Agency,Washington, DC, USA* **2015**.

(4) Goss, K. U. Predicting the Equilibrium Partitioning of Organic Compounds Using Just One Linear Solvation Energy Relationship (LSER). *Fluid Phase Equilib.* **2005**, *233*, 19–22. https://doi.org/10.1016/j.fluid.2005.04.006.

(5) Ulrich, N.; Endo, S.; Brown, T. N.; Watanabe, N.; Bronner, G.; Abraham, M. H.; Goss, K.-U. *UFZ-LSER database v 3.2.1*. Helmholtz Centre for Environmental Research-UFZ, Leipzig, Germany. http://www.ufz.de/lserd.

(6) Endo, S.; Watanabe, N.; Ulrich, N.; Bronner, G.; Goss, K.-U. *UFZ-LSER database v 2.1*. Helmholtz Centre for Environmental Research-UFZ, Leipzig, Germany.

(7) Allendorf, F.; Berger, U.; Goss, K. U.; Ulrich, N. Partition Coefficients of Four Perfluoroalkyl Acid Alternatives between Bovine Serum Albumin (BSA) and Water in Comparison to Ten Classical Perfluoroalkyl Acids. *Environ. Sci. Process. Impacts* **2019**, *21*, 1852–1863. https://doi.org/10.1039/c9em00290a.

(8) Allendorf, F.; Goss, K. U.; Ulrich, N. Estimating the Equilibrium Distribution of Perfluoroalkyl Acids and 4 of Their Alternatives in Mammals. *Environ. Toxicol. Chem.* **2021**, *40*, 910–920. https://doi.org/10.1002/etc.4954.

(9) Endo, S. Intermolecular Interactions, Solute Descriptors, and Partition Properties of Neutral Per- and Polyfluoroalkyl Substances (PFAS). *Environ. Sci. Technol.* **2023**, *57*, 17534–17541. https://doi.org/10.1021/acs.est.3c07503.

(10) Endo, S.; Hammer, J.; Matsuzawa, S. Experimental Determination of Air/Water Partition Coefficients for 21 Per- and Polyfluoroalkyl Substances Reveals Variable Performance of Property Prediction Models. *Environ. Sci. Technol.* **2023**, *57*, 8406–8413. https://doi.org/10.1021/acs.est.3c02545.

(11) Xiang, Q.; Shan, G.; Wu, W.; Jin, H.; Zhu, L. Measuring Log Kow Coefficients of Neutral Species of Perfluoroalkyl Carboxylic Acids Using Reversed-Phase High-Performance Liquid Chromatography. *Environ. Pollut.* **2018**, *242*, 1283–1290. https://doi.org/10.1016/j.envpol.2018.08.009.

(12) Endo, S.; Brown, T. N.; Goss, K. U. General Model for Estimating Partition Coefficients to Organisms and Their Tissues Using the Biological Compositions and Polyparameter Linear Free Energy Relationships. *Environ. Sci. Technol.* **2013**. https://doi.org/10.1021/es401772m.
